# Bioengineered biochar as smart candidate for resource recovery toward circular bio-economy: a review

**DOI:** 10.1080/21655979.2021.1993536

**Published:** 2021-12-11

**Authors:** Hong Liu, Vinay Kumar, Vivek Yadav, Shasha Guo, Surendra Sarsaiya, Parameswaran Binod, Raveendran Sindhu, Ping Xu, Zengqiang Zhang, Ashok Pandey, Mukesh Kumar Awasthi

**Affiliations:** a College of Natural Resources and Environment, Northwest A&F University, Yangling, PR China; b Department of Biotechnology, Indian Institute of Technology(IIT) Roorkee, Roorkee, India; c State Key Laboratory of Crop Stress Biology in Arid Areas, College of Horticulture, Northwest A & F University, Yangling China; d Institute of Tea Science, Zhejiang University, Hangzhou, China; e Key Laboratory of Basic Pharmacology and Joint International Research Laboratory of Ethnomedicine of Ministry of Education, Zunyi Medical University, Zunyi, Guizhou, China; f Microbial Processes and Technology Division, CSIR-National Institute for Interdisciplinary Science and Technology (CSIR-NIIST), Thiruvananthapuram, India; g Centre for Innovation and Translational Research, CSIR-Indian Institute of Toxicology Research, Lucknow, India

**Keywords:** Circular bio-economy, biochar, engineered process, sustainable technologies, resource recovery

## Abstract

Biochar’s ability to mediate and facilitate microbial contamination degradation, as well as its carbon-sequestration potential, has sparked interest in recent years. The scope, possible advantages (economic and environmental), and future views are all evaluated in this review. We go over the many designed processes that are taking place and show why it is critical to look into biochar production for resource recovery and the role of bioengineered biochar in waste recycling. We concentrate on current breakthroughs in the fields of engineered biochar application techniques to systematically and sustainable technology. As a result, this paper describes the use of biomass for biochar production using various methods, as well as its use as an effective inclusion material to increase performance. The impact of biochar amendments on microbial colonisation, direct interspecies electron transfer, organic load minimization, and buffering maintenance is explored in detail. The majority of organic and inorganic (heavy metals) contaminants in the environment today are caused by human activities, such as mining and the use of chemical fertilizers and pesticides, which can be treated sustainably by using engineered biochar to promote the establishment of a sustainable engineered process by inducing the circular bioeconomy.

## GRAPHICAL ABSTRACT

**Figure:**
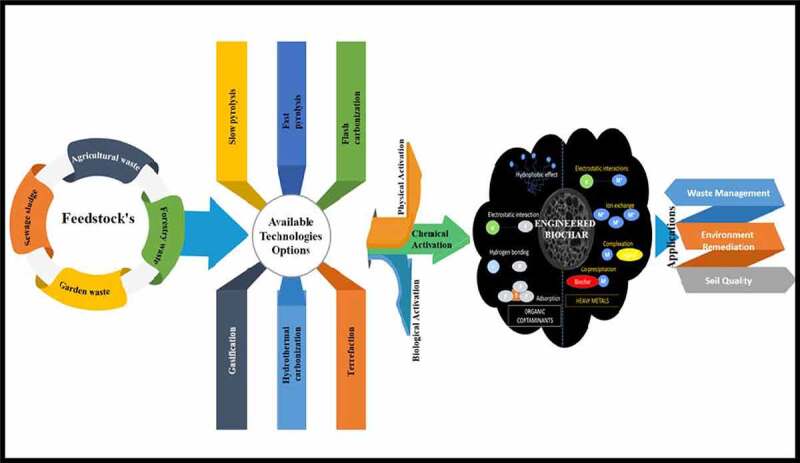

## 1. Introduction

Rapid exponential growth in resource consumption and exploitation has resulted in scarcities of resources. The current energy system enjoys an unsustainable approach and presents an energy system trajectory moving away from sustainability goals [1]. Bioengineered biochar is a heterogeneous bioproduct generated from carbonization thermochemical conversion of feedstock in an oxygen-limited environmental condition, which is further modified by various methods to improve the utilization and increase the overall efficiency [2]. Circular bio-economy [3] is a broad and fluid concept with many definitions, yet all leading to single goal that talk about greater resource efficiency with sustainability [4,5]. The concept is still developing and tranquilly required fundamental research, in the precise utilization of engineered biochar for sustainability goals [6]. Biochar has a huge untapped potential to substitute unfriendly techniques to address the climate change issues, environmental impact and establish a bio-circular economy model [7]. The utilization of engineered biochar in the environment and energy sector is an operative tool to reduce waste and increase participation in the circular bio-economy.

With the rapid development of modern society, a large amount of organic solid waste has been produced [8]. Because landfills occupy a large area and produce a large number of auxiliary pollutants [9,10], many areas are beginning to use compost to treat pollutants and recycle solid waste [11,12]. And some studies have found that the addition of biochar can improve the efficiency of composting effective measures [13]. Biological waste to energy technology is a feasible way to treat waste and produce energy [14]. Resource recovery from waste is an emerging trend towards the utilization of biochar for achieving the circular bio-economy goals [15,16]. The concept encompasses several strategies, including energy solutions, reuse and recycling, and design for durability. However, the concept is still growing and not adopted for many industrial applications. The establishment of resource recovery from feedstock waste required the implementation of novel concepts and innovative ideas. The multi-dimensional role of engineered biochar in energy and environmental perspective endows its great value for resource recovery [17]. Despite concrete strength, the research of resource recovery from waste by biochar utilization is debated [7]. Biochar has been extensively exploited for various approaches from the last decade. Recent studies showed that strategically modified biochar has great potential for large-scale application in various fields. A case study from Singapore showed that organic waste generated from the horticulture sector has potential strength to participate in the circular economy by on-site use of biochar valorized via gasification. Further research emphasized that organic waste mass was reduced up to 95% through gasification by conversion to biochar. The case study detailed that biochar produced from organic waste can substitute the use of peat moss and effectively utilize it for the cultivation of various commercial horticulture crops including Pak-Choi, lettuce and pansy [18]. In another case, wood waste-derived biochar was used to upgrade biogas production in anaerobic digestion. Food waste digestion was carried out with biochar to upgrade biogas production and a further mixture of biochar and AD generated waste was used to grow various vegetable crops [19]. Similarly, the use of biochar in circular economy was conceptualized in agriculture sectors. It was evidenced that circular economy efficiency can be used by utilization of biochar in N, P, and K cycles [20]. The use of biochar for the replacement of chemical fertilizers can help in promoting organic farming and a sustainable farming system. Moreover, the use of biochar as a replacement for growing media can effectively boost resource recovery and contribute to the bio-circular economy [21].

Here our review offers a state of the current basic strategic approach for utilization of bioengineered biochar for resource recovery and the possibilities for use of biochar in circular bio-economy. This review will provide a unique approach that can help think tanks to frame strategies aiming for clean technology and energy solution by utilizing biochar engineering.

## 2. Strategies for biochar bioengineering

Rapid development and overpopulation of industries and lifestyles is directly associated with waste generation, which had an adverse impact on the environment. Hence, there is a need towards a holistic approach to sustainable waste recycling. Biochar production is a simple technology suitable for all developed and developing countries [22]. Engineering the physicochemical properties of biochar will improve effective waste recycling. The basic properties including surface area, pore space, functional group, and pore structures can be engineered by some biological, physical and physical methods [23,24]. Several microorganisms are reported to improve the biochemical properties of biochar. These microbes will bind by direct or indirect strategies to engineer the properties of biochar [25]. Bioengineering methods are generally post-modification techniques. The application of targeted microorganisms will modify biochar either by increase in surface area, colonization, and development of biofilm. The common strategy adopted is employing beneficial microbes for surface coating of biochar for improving remediation performance [26,27]. This will enhance the adsorption efficiency of different pollutants. Some microbes are known for bioremediation of heavy metals [25,26,28]. Dalahmeh et al., 2018 demonstrated that filter designed of biochar active biofilm absorbs and degrades >98% carbamazepine in comparison to sand active biofilms [29]. It also adsorbs pharmaceutical compounds including metoprolol, carbamazepine, caffeine, and ranitidine that were degraded effectively from sewage water. The utilization of consolidate obtained by amalgamation of biologically treated feedstock (digestate) and biochar were also used as bioengineered biochar [27]. The results indicate that anaerobic digestion-mediated modifications lead to an increase in surface area, anion exchange capacities, pH and adsorption potential [30]. These engineered biochar exhibited super adsorption of heavy metals from soil and water. An integrated strategy of AD and biochar engineering can provide a better alternative and increase economic feasibilities by harvest of biogas and biofertilizers [25,31,32].

## 3. Application of engineered biochar in different fields

Engineered biochar can be prepared from various resources such as wood wastes [33], crop residues [34], animal wastes [35], peanut shells [36], algal biomass [37], sawdust [38], sugarcane bagasse [39], tea waste, [40] and biosolids [41]. The addition of biochar has tremendous advantages [42]. These include carbon sequestration [43], soil quality improvement [44], modifying physiological characteristics of soil [45], enhancing mycorrhizal associations [46,47], improving soil structure [48], altering bioavailability, improving nutrient cycling [49], and soil nutrient-holding capacity [43,50,51]. Biochar generally has excellent properties such as high stability, well-developed pore structure and abundant surface functional groups [52]. Biochar has a high affinity for nutrient absorption, and it prevents nutrient leaching, improves nutrient conversion [53], leading to improved crop yield. Biochar has been reported to alter the crop yield in the present as well as in the absence of fertilizers [54]. Biochar can reduce the adverse factors in traditional composting and improve the efficiency of composting [55]. Biochar can be prepared to utilize various feedstock’s and to carry out a specialized function; biochar must be improved in terms of affinity, porosity, binding affinity, functional groups and reusability. In recent years, various approaches have been applied to design sustainable and effective biochar for environmental applications. There are three areas that require immediate attention to develop engineered biochar. The developed biochar can be used in greenhouse gas emissions, compost remediation, and improvement of microbial populations.

### 3.1. Role of biochar in the reduction of greenhouse gas emission

The major greenhouse gases are methane (CH_4_), carbon dioxide (CO_2_), and nitrous oxide (N_2_O). The agriculture sector contributes to a major part of greenhouse gas emissions [56]. Nitrogen fertilizer contributes to about 70% of N_2_O emissions. As per the UNFCC Paris Agreement, the global greenhouse gas emissions must be reduced to about 6% yearly and to regulate the global temperature elevation to below 2°C [57,58]. Several methods are available for the removal of greenhouse gases [59]. Several studies reported the use of agricultural wastes and residues as a tool for mitigation of greenhouse gas emissions. In recent years, biochar application has been considered an effective method to reduce greenhouse gas emissions [60–62] because it has a good adsorption capacity for greenhouse gases and ammonia [63]. It has been known to alter the abundance of greenhouse gases in different environments. For application in greenhouse gas mitigation, biochar can be derived from softwood [64], food waste [59], biochar-fertilized chernozems [65], biochar manure mixtures [66], activated biochar pellet fertilizers [67], straw [68,69], walnut and coconut shells [50], and nZVI-biochar nano-composite [70].

Biochar can be prepared from straw and can be applied to the crops. In this regard, a study was conducted where the effects of amending the biochar derived from straw were evaluated for emissions of carbon dioxide, methane, and nitrous oxide [71]. The study demonstrated that biochar application in combination with urea increased methane emissions by ten-fold as compared to urea alone. Also, there was an enhancement in the CO_2_ uptake, but N_2_O emission was decreased. This CO_2_ uptake leads to a significant decrease in global warming potential. A study was performed in the soil to analyze the effect of biochar application on greenhouse gas production rate, soil physicochemical properties and the microbial community at varying temperatures [72]. The results indicate that with a rise in temperature, a higher greenhouse gas production was observed [73]. Therefore, a direct correlation was observed between temperature and greenhouse gas production and biochar application. The emission of greenhouse gases on the one hand led to the reduction of overall microbial communities and, on the other hand, supports the growth of some specific microbial communities such as *Actinobacteria* and *Acidobacteria*. Biochar application caused a reduction in soil N_2_O production, CH_4_ consumption and an increase in total carbon content [74].

A recent study was focused on the determination of cumulative N_2_O-N, CO_2_-N, and CH_4_-C emissions on the application of pyrogenic-C biochar [65]. The results demonstrated that cumulative CH_4_-O and N_2_O-N emissions were independent of biochar influence and addition of NP fertilizer. But cumulative CO_2_-C emissions were affected by biochar amendment with -NP fertilizer and varied non-linearly. The study demonstrated that the two types of soils used in the study, namely sandy loam and clayey had very close NO_2_-N and CO_2_-C fluxes irrespective of NP fertilizer. The methane flux was reported to be low in the incubation time. It was also observed that cumulative CO_2_-C was affected by soil fertilizer interaction. The study concluded that biochar amendment on soil partially benefited Chernozems. Reports evidenced that the CO_2_-C flux reduction is affected by biochar applications [43]. There are several factors that affect the CO_2_-C emissions. These include initial soil properties, biochar feedstock and N-fertilizer application [43]. Multivariate redundancy analysis was performed to analyze the relationship between factors affecting soil nutrients cycling (NP-fertilization, biochar application, and soil texture) and factors affecting environmental performance such as water-extractable organic matter pool, nutrient availability, and cumulative greenhouse gas emissions [65]. Global meta-analysis studies were also conducted on greenhouse gas emissions [75,76]. A meta-study demonstrated that the application of biochar causes a reduction in CH_4_, N_2_O and NH_3_ emissions [77]. Recently, a study was focused on the analysis of commercial biochar stability in combination with clinoptilolite for the reduction in gas emissions [78].

There are various parameters that need to be considered before biochar applications. While considering the effects of biochar amendments on greenhouse gas emissions, it is an immediate requirement to understand the global warming potential of these emissions [71]. Majority of the research are on the application of fresh biochar. However, it has been observed that with the aging of the biochar, its properties change, which leads to variable response. It has been observed that as compared to fresh biochar, aged biochar has different responses [72,79]. Fresh biochars are reported to be more efficient in terms of adsorption of greenhouse gases in comparison to aged biochar. Aged biochar showed reduced efficiency in adsorption of greenhouse gases and also induced the leaching of small particulate and dissolved components in soil. The soil properties and microbial colonization in soil amended with biochar change with time which in turn affects the soil nitrogen transformation.

It has been observed that the addition of straw to the soil improves nutrients availability and physical properties [80]. Application of straw to the soil has advantages such as it reduces the emission of pollutant associated with the straw burning and it helps to enhance the carbon storage capacity of the soil [68]. Regardless the beneficial aspects associated with the straw addition to the soil, there are some reports that mention that straw addition causes increased emissions. For example, studies conducted on farmlands demonstrated that N_2_O emissions were increased on the application of straw [80,81]. The emissions of N_2_O and CH_4_ were increased significantly after the addition of either rice or wheat straw. It is known that straw and biochar application can alter the consumption as well as production of CH_4_ and N_2_O. A study was conducted where the application of biochar in association with intermittent aeration and tidal flow was observed [69]. The study results demonstrated that biochar addition reduced the N_2_O and CH_4_ fluxes, which supports the increased abundance of nosZ and mcrA genes. This led to a decrease in global warming potential by 55.8%. Moreover, it resulted in 41.3% total nitrogen removal. The study demonstrated that the addition of biochar along with tidal flow led to nitrogen removal, but it also led to an increase in greenhouse gas fluxes.

In constructed wetlands, both aerobic and anaerobic conditions are prevalent. Therefore, they are considered as the hotspot for N_2_O and CH_4_ production. This happens due to a high pollutant load [69,82]. It is estimated that greenhouse gas emissions from constructed wetlands will increase significantly due to their wide applications. In constructed wetlands, substrate plays an important role in determining global warming potential because it provides a growth medium for the organisms [83,84]. Biochar has been known to improve nitrogen removal in constructed wetlands [85]. Studies have reported that addition of biochar can reduce the global warming potential of gravel-based constructed wetlands [83,86]. To understand the mechanism of greenhouse potential of biochar-mediated emissions in constructed wetlands, dissolved oxygen can play an important role. Dissolved oxygen can control various microbial reactions associated with greenhouse emissions and pollutant removal [87]. In constructed wetlands, the major problem is the low release of dissolved oxygen due to limited atmospheric re-aeration. Therefore, the oxidation process is restricted, and thereby the NH_4_^+^ removal by nitrification is affected [88]. Therefore, supplying the oxygen periodically in constructed wetlands to perform alternating aerobic and anaerobic processes for total nitrogen removal is a requirement. In constructed wetlands, microbes release greenhouse gases by biodegradation and biotransformation. The composition of these microbes, their abundance and their activities are the indicators of greenhouse gas emissions [89]. It is also known that nitrifying, denitrifying, and methanogenic bacteria are associated with the production of greenhouse gases [90–92].

It has been known that nitrous oxide (N_2_O) has a considerable impact on ozone layer depletion. It has several times more global warming potential than other greenhouse gases. Of the total global anthropogenic emissions, NO and N_2_O emissions contribute to 10% and 59%, respectively [79]. In recent years, there is a tremendous increase in use of nitrogen fertilizers. Nitrification is a crucial step for the production of NO and N_2_O. The two steps in nitrification are ammonia oxidation and nitrite oxidation. Biochar application results in soil pH increase. This increase in soil pH is due to the biochar’s ability to absorb exchangeable acidic cations. The change in pH plays an extraordinary role in controlling the structure and abundance of ammonia oxidizers, which finally affects the production of NO and N_2_O [93]. However, several studies were focused on the application of fresh biochar [59]. However, studies focusing on the application of aged biochar are lacking. In this context, a few studies utilized and compared the effect in soil for fresh and aged biochar [79,94]. It was observed that aged biochar had lost the ability for N_2_O emissions as compared to fresh biochar.

The results from a study suggested that the application of biochar has a considerable effect on N_2_O production in soil [72]. The study suggested that there could have been several abiotic and biotic mechanisms responsible for soil N_2_O production. The first mechanism is that after biochar application, microbial activities could have stimulated inducing the N immobilization. This will be led to restricting the substrate availability to produce N_2_O. The addition of litter to the soil also had a negative N_2_O production effect. The second mechanism is that the application of biochar suppressed the autotrophic nitrification processes [94]. In acidic soil, this will reduce the yield of ammonia-oxidizing bacteria [79]. Due to the above reasons, there will be an overall reduction in N_2_O production. The third mechanism is the suppression of anaerobic denitrification in moist and acidic soils [95]. The fourth mechanism is that N_2_O redox reactions can be mediated by biochar as a chemical catalyst [72]. Moreover, biochar may restrict the availability of N by altering the inorganic N adsorption. The production of N_2_O will be reduced significantly, if there is a reduction in the availability of the substrate. In addition to the direct effect, biochar can increase soil pH, thereby improving soil aeration. The increased soil pH facilitates the absorption of NH_4_^+^ from the soil, limiting the denitrification and nitrification processes [71]. Enhancement of soil aeration leads to CH_4_ oxidation and a decrease in CH_4_ production.

CH_4_ is produced by methanogenic bacteria in anaerobic conditions and it is influenced by fertilization in the presence of organic carbon [96]. In agricultural soil, the addition of crop residues leads to the increment of specific methanogenic bacterial populations, which leads to increased CH_4_ production. Enhanced CH_4_ production acts as a stimulator for methanogenic bacteria growth and contributes to CH_4_ oxidation. For CH_4_ production, pmoA gene encoding methane monooxygenase and the mcrA gene encoding methyl coenzyme M reductase are considered as phylogenetic markers [90,97]. Although several studies have demonstrated the effect of greenhouse gases but in most of the studies, climate change associated with plant CO_2_ uptake has been underestimated. The addition of crop residues to the plants and greater photosynthesis benefit plant growth [98]. The biochar amendment has a varying rate of greenhouse gas emissions. It has been observed that CO_2_ emissions were not significant in soil [99].

### 3.2. Role of biochar amendment for remediation of compost

Engineering biochar approaches decrease the bioavailability and mobility of contaminants [100]. This can be achieved by escalating the number of the binding site and introducing the microorganisms with properties to degrade contaminants. In this way, biochar can be invented in such a way that it can have both a high number of binding sites as well as microbial degradation activities. The pristine biochar can be modified to improve the required chemical, physical and biological properties [23,101,102]. Approaches such as biological methods provide diversity to the biochar [103,104], while chemical methods provide improved functional groups [105]. Physical modification of biochar can be performed by the use of electrochemical, ultrasound, plasma, steam and thermal methods. Physical methods improve the biochar physicochemical properties such as pore volume, surface area, ash content, aromaticity, pH, and polarity [105,106]. In biological engineering, the digestate produced from anaerobic digestion is amalgamated with biochar [107–109]. Another biological approach is to inoculate promising contaminant degrading microbes on biochar surfaces [26].

There are various methods available for biochar production for remediation applications. These include hydrothermal carbonization, pyrolysis, microwave carbonization, gasification, flash carbonization, torrefaction, plasma cracking and laser cracking. Three methods, gasification, pyrolysis, and hydrothermal carbonization, are used more than others [110]. But pyrolysis is considered most efficient because it is easy, requires less energy and cost-effective [111–113]. For other techniques such as torrefaction, gasification, and carbonization which are used majorly for solid fuel, bio-oil and synthetic gas yield [114]. Recently, studies on pyrolysis are focused on the alteration of traditional methods and the development of new one. The purpose is to produce engineered biochar with improved properties and mitigate the issues of environmental contamination during their application. The new, improved pyrolysis method improved the biochar properties, such as higher carbonization and reduction in functional groups such as oxygen [115]. Low oxygen functional groups are also produced in steam-assisted pyrolysis [116]. Heavy metals can be removed using steam and microwave-assisted pyrolysis [117]. The advantage of reducing the oxygen-containing functional group is that it leads to an interaction between polyaromatic hydrocarbons and biochar via electrostatic interactions [115]. There are various physicochemical properties of biochar, including specific area, yield, pore structure, cation exchange capacity and a number of functional groups [118].

Various factors such as pyrolysis temperature, feedstock material, heating rate, reaction atmosphere and residence time affect the pyrolysis process and hence the biochar physicochemical properties. It has been observed that the stability and physicochemical properties of biochar can be modified with an alteration in the proportion and composition of lignocellulose [119]. Reaction temperature can be considered as an important parameter influencing the biochar physicochemical properties. An increase in pyrolysis temperature can result in an increment in pore diameter, specific surface area, ash content and pH [114,120]. A reduction in the oxygen-containing functional group is observed on high pyrolysis temperature. Biomass materials can be maintained at low pyrolysis temperatures [120,121]. Pyrolysis can be performed using two methods, namely slow and fast pyrolysis. Slow pyrolysis involves a long period of heating in hours at 400–600°C without oxygen [122]. Slow pyrolysis produces high yield of biochar [114]. In contrast, fast pyrolysis takes place in a very short period of time in milliseconds or seconds at a higher temperature 300–700°C [123].

A study demonstrated that the application of sugarcane bagasse biochar on *Brassica chinesis* L. resulted in a decrease in bioavailability of heavy metals such as Cd, Pb, and Cu [124]. In another study conducted at the mesocosm scale, it was observed that application of biochar (5% w/w) resulted in a decrease in total petroleum hydrocarbon [125]. When the soil was amended with biochar (2.5%) in a field experiment, it resulted in significant polyaromatic hydrocarbon reduction [126]. Biochar prepared from rice straw, and peanut vine was amended on latosolic soil, which resulted in a reduction of exchangeable Cd [127]. Biochar prepared from agriculture residue and applied on loamy soil resulted in mitigation of expendable Cd and Pb [128]. When pecan shell was used to prepare biochar, it resulted in adsorption of a very high Pb concentration [129]. Biochar derived from vegetable wastes resulted in immobilization of a very high Pb concentration in sandy loam [130]. A reduction in pyrene concentration was observed on the application of biochar derived from olive pomace [131]. Application of rice straw-derived biochar in sandy soil resulted in a significant polyaromatic hydrocarbon reduction [99,132,133]. Biochar derived from maize straw resulted in a higher polyaromatic hydrocarbon degradation [134,135].

Digestate can be used as a tool to remove the pollutant from the soil. Digestate acts as a biostimulant for the removal of contaminants. Digestate, a rich source of micronutrients, increases microbial activities and population. It is a rich nutrient source containing a diverse and dense population of microbes [27]. The consequent increase in the bacterial population and activities lead to the removal of the contaminants [103,104]. The anaerobic digestion process is required to produce digestate from agricultural food wastes and organic fraction of municipal solid wastes. Digestate is composed of three parts, namely: microbial organisms, undigested feedstock, and microbial metabolites. It can further be classified as solid and liquid digestate [136]. The utilization of agricultural food wastes and organic fraction of municipal solid wastes in digestate preparation promotes the concept of a circular economy [137]. Digestate nutrients are responsible for promoting microbial activity, diversity, and abundance [103,104]. Biochar in combination with digestate increases the contaminants removal efficiency. A significant reduction in removal of Zn and Cd was observed when cow manure biochar was applied with digestate in a French technosol [138,139]. In a study, it was observed that addition of digestate along with bacteria immobilized biochar resulted in 95% petroleum hydrocarbon degradation in soil [108,109]. A similar study conducted on sandy soil and clay-rich soil using digestate and immobilized bacteria resulted in a higher petroleum hydrocarbon degradation than in controls [103,104]. There are various advantages of using bioengineered biochar technologies for the removal of contaminants. The advantages are environment friendly, sustainable, low cost, capable of immobilizing pollutants, able to improve the soil quality, water retention, microbial growth-promoting activities, and carbon sequestration potential [125,128].

**Figure 1.**
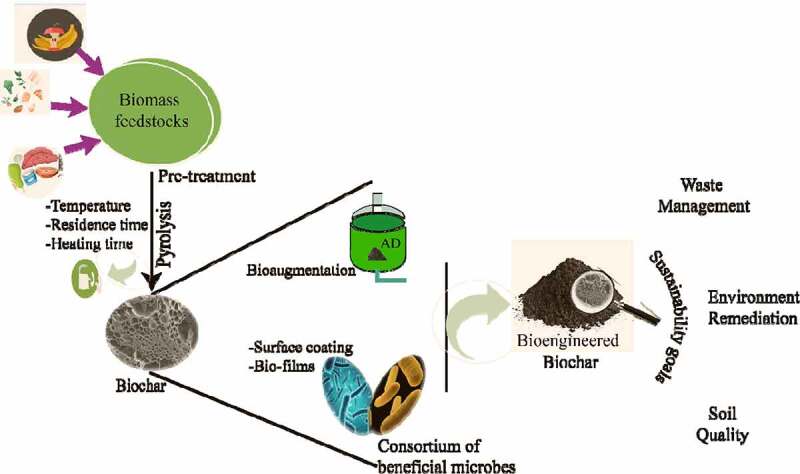
Bioengineering methods of biochar and sustainability goals

Microbes can be inoculated and immobilized on biochar to develop an effective tool for soil bioremediation. In the methods, the immobilized microbes release the enzymes responsible for detoxification of the contaminant. These enzymes act upon the target substrates in the environment to break down into simpler compounds. The polyaromatic hydrocarbons are mineralized to CO_2_ and H_2_O [27]. Microbes sequester the pollutants either through bioaccumulation or adsorption process. The microbial cell walls contain negatively charged functional groups such as amino, carboxylate, sulfate, hydroxyl, phosphate, and amine, which can bind to metal ions and thereby sequester them from the soil [27]. In a study, a bacterial strain NT-2 (*Pseudomonas* sp.) which was tolerant to Cd-Cu, was used with biochar. It was observed that biochar as a carrier was responsible for reducing the bioavailability of Cd^2+^ and Cu^2+^ in soil. The biochar enhanced the soil enzyme activity [26]. Phosphate solubilizing bacteria was used to increase the Pb^2+^ immobilization in sludge and rice biochar. It was observed that colonization of bacteria on the biochar leads to a higher Pb^2+^ reduction. In another study, *Bacillus subtilis* was used for soil remediation of heavy metals using pig manure and corn straw biochar. The results indicated that biochar amended with bacteria reduced the heavy-metal concentrations in the lettuce-edible parts [140]. In a decontamination study, a consortium of three bacteria amended with biochar was used to remove Cd and U from soil. The results demonstrated a significant reduction in Cd and U [141]. There are various mechanisms through which biochar interacts with pollutants and heavy metals. A list of various interactions between biochar and pollutants is presented in the (Figure 1).

In a study, biochar resulted in decreased As bioaccumulation in plants [142]. Biochar derived from sawdust resulted in enhanced As mobility [143]. Rice straw-derived biochar resulted in decreased availability of Cd and Pb, while it resulted in increased availability of As [144,145] and strong interaction with heavy metals [81]. Chicken manure-derived biochar resulted in decreased metal mobility [120]. Rapeseed cake biochar resulted in enhanced binding capacities for Cu [146]. In the removal of metal ions, the dissolved organic matter-derived biochar chelates with metals [147,148]. The chelating effect influences bioavailability, associated toxicity, and metal speciation [81]. In complexation with potentially toxic elements and dissolved organic matter, it was observed that complexation was hampered by water hardness and soil salinity [149]. This happens due to competition among cations for the complexation sites. It has been observed that dissolved organic matter derived from biochar had a tendency to bind with Cd with carboxyl and phenolic group [81]. After forming a complex between Cd and dissolved organic matter, the complex mobility is hindered due to microbial degradation activity [144]. It is observed that the functional group responsible for binding with Pb was N = O and -NO_2_ [150]. After complex formation between metals and dissolved organic matter, it was observed that the availability of Pb, Zn and Cd was decreased [123,148]. It has also been reported that the dissolved organic matter-based biochar increases the pH value of soil [148]. Biochar-based dissolved organic matter is responsible for maintaining the redox potential of soil which enhances the soil microbe activities. The enhanced activity will affect the contaminant bioavailability and its accumulation in plants [142]. The oxyanions pollutant’s bioavailability can be increased by various mechanisms. In soil, biochar can form complexes with As and Fe [143,151]. The valence ions in the oxy anions in arsenic and chromium may be affected by the electron transfer process. Biochar can act as both an electron acceptor as well as an electron donor. This is because of its heterogeneous components [152]. The electron transfer capability for Cr (VI) reduction of biochar is exhibited by a high content of aromatic moieties and quinone [153]. In a study, it was observed that walnut-derived biochar was capable of potassium chromate oxidation [154]. Cr (VI) reduction in soil was observed with chelation of soil Fe(II) and Fe(III) through the photoreduction process by biochar [151]. Recently, several studies have demonstrated the biochar remediation mechanisms [112,141,155]. In the case of inorganic pollutants, including heavy metals, the remediation mechanism is based on the valence state of the metal ion [156].

Biochar-based dissolved organic matter can participate in controlling the destiny of the organic contaminants in the soil. A study had reported that biochar could alter the soil’s hydrophobic surface and physicochemical properties, thereby hindering the mobility of pesticides [157]. Biochar exhibits photocatalytic activity by absorbing light and formation of reactive oxygen species for the degradation of organic contaminants. It was observed that the biochars having a higher aromaticity have a higher photoreactivity as compared to with other groups [158]. Biochar can be used in combination with nano-zero-valent iron (nZVI) to provide stability [159]. The advantage of using iron-based biochar composites is that it exhibits excellent performance for contaminant remediation [160–162]. Single heavy metal removal by biochar immobilization is more popular as compared to multiple heavy metal immobilizations [163]. A study was focused on Pb (II) sequestration by biochar catalyst derived from sludge [164].

Biochar has shown a great potential for pesticide remediation in soil. In soil, biochar has been reported to reduce the concentration of dimethoate [165]. Biochar in a combination of metal nanoparticles are also known to have immense application in the area of remediation [166]. Biochar had been reported for remediation of soil contaminated with pesticides such as imazapyr, chlorpyrifos, and thiamethoxam [167–169]. Sometimes, a very high sorption capacity of biochar is observed so that a higher level of pesticide accumulation is observed [45,170]. In agricultural soil, biochar caused a reduction of thiamethoxam in Chinese chive [169]. In a study, wood waste-derived biochar application reduced the chiral pesticide metalaxyl uptake in lettuce [171]. The biochar increased the soil bacterial community, causing an increase in the population of the pesticide degrading bacteria and a shift in bacterial community. In another study, biochar application resulted in increased cadmium remediation, hindered nitrous oxide production and alteration in rhizosphere microbial activity [172]. There are various interactions by which pesticides can be retained on solid surfaces such as biochar. These interactions include hydrogen bonding, hydrophobic interactions, Vander Waals forces and covalent bonds [173,174]. Sorption is an important phenomenon that plays a crucial role in determining the fate of pesticides. It was observed that pesticides fluoridone and norflurazon sorption might take place due to Vander Walls interactions.

### 3.3. Effect of biochar amendment on microorganisms during composting

Biochar modifies various parameters in soil and hence the growth conditions for the microbe. Due to its unique properties, biochar can change the microbial community associated with soil. Various functional groups present in the biochar help in facilitating activities such as greenhouse gas emissions, remediation of toxic compounds such as heavy metals and alteration in microbial communities. It can improve microbial growth, adsorption capacity and soil aggregate stability [99]. Biochar has been known to alter the soil microbe’s soil activity and ultimately modifying the soil microbial community structure [175]. There are several parameters that need to be considered before starting the analysis of the soil bacterial communities [176].

Biochar has been amended in various soils using different methods. In a study, biochar-assisted immobilization of bacterial strain NT-2 resulted in improved soil microbial community [26]. In another study, biochar application in soil demonstrated that it did not disturb the microbial community but carried out Cd remediation in soils [172]. In a different study, when peanut shell biochar was applied to the soil, it resulted in a reduction of potentially toxic elements such as Ni, Cd, As, Pb and Cr [177]. It has been observed that when biochar is combined with fertilizers, it shows a good remediation potential for soil contaminated with crude oil [178]. A recent study resulted in increased microbial activities in an organic carbon deficient arid soil [179]. Dissolved organic matter released from biochar acts as a major carbon source for the growth of microbial communities. Therefore, it supports the microbial activities and population. A study reported that when microbes were cultured in a dissolved organic matter derived from wheat straw, it resulted in stable microbial growth [180]. The microbial growth leads to enhanced microbial respiration. The increased microbial activity will support the biochar humification process and release of organic matter [120]. Application of biochar derived from carbon feedstocks such as sorghum, wood, food waste, anaerobic digestion residues, and chicken manure [181] results in increased soil dissolved organic content, high bacterial proliferation, and increased fungi communities [120]. The application of straw manure resulted in increased microbial abundance [182].

The soil microbial activity and population is influenced by the application of biochar [123,183,184], and soil functioning is affected by soil microbial population [120,124]. There are various methods available to analyze the level of microbial abundance biochar applied to the soil. These methods include fumigation extraction [184], phospholipid fatty acid extraction [185], genomic DNA extraction [130] and increase microbial reproduction [186,187]. The change in the microbial populations varies in relation to microbial diversity. Biochar application, on the one hand, can increase the microbial population of specific genera such as *Gemmatimonadetes, Actinobacteria, firmicutes* and *Proteobacteria*. On the other hand, it can decrease the microbial population of other microbes such as *Acidobacteria* and *Chloroflexi* [185]. Temperature and climate variations play an important role in microbial population growth and diversity. In higher temperatures, an increase in microbial biomass was observed [72]. Biochar-mediated microorganism activity is influenced by climate change [188]. Studies were conducted to analyze the effects of biochar in temperate and tropical climates on a long-term basis [180,189,190]. Some reports have investigated the effects of biochar in boreal agricultural clay soils [64,191].

In soil, the rhizosphere plays an important role in nutrient cycling. The rhizosphere is the area around the plant root where microbes are accumulated to carry out a diverse array of chemical and biological processes. Studies were conducted to determine the fate of microbes associated with the rhizosphere in different soils [192,193]. It was observed that biochar is advantageous because it can enhance plant performance and change the plant rhizosphere microbial communities, thereby suppressing plant pathogens. A study analyzed the effects of biochar application on the bacterial community in tomato rhizosphere in calcareous soil. The special feature of calcareous soil is that it has a neutral or alkaline pH [193]. The study results demonstrated that the amendment of biochar resulted in soil pH enhancement, increment in carbon concentration, and increased vegetative productivity in tomato plants. In the context of the bacterial community in the rhizosphere, no significant changes were observed. The major dominant phyla were *Proteobacteria, Actinobacteria, Acidobacteria, Bacteroides, Chloroflexi* and *Gemmatimonadetes*. The study also demonstrated that the taxonomic composition between biochar treatments and control was similar. Overall, the result demonstrated that the amendment of biochar has a limited effect on the rhizosphere bacterial community. Biochar can absorb the plant root exudates, and thereby it affects plant-derived C utilization by the microbes. A study used ^13^CO_2_ to analyze the rhizosphere bacterial communities’ abundance. The study demonstrated that the application of biochar modified the bacterial community actively engaged in plant-derived C assimilation. It also modified the shape and rhizosphere community [192]. As compared to the control soils, a higher abundance of bacterial species was observed in biochar applied soils [194]. The relative abundance of *Bacteroides* and *Firmicutes* was increased as compared to the *Proteobacteria* members in biochar applied soils. In a different study, biochar application increased the abundance of a particular genus of bacteria such as *Haliangium* [195] and *Asticcacaulis* [196].

Phosphate solubilizing bacteria makes P available in a form that can be easily accessed by the plants. In biochar-amended soils, the abundance of phosphate-solubilizing bacteria was observed, which may be due to a microbial community shift after biochar applications [197]. Biochar application can modify the bacterial activities such as soil phosphatase and mineralization of organic P [198]. A significant increase in the P-solubilizing bacteria was observed when soil was amended with biochar from leaves of *C. lanceolat*e [175]. The application of straw biochar on soil has resulted in increasing the abundance of inorganic P-solubilizing bacteria [199]. In a study, an increment in the bacterial population was observed after biochar and straw application [68]. The study also demonstrated that the addition of biochar to the soil resulted in a reduction in denitrifying bacteria and a decrease in ammonia-oxidizing archaea. A study reported biochar application shifted the microbial community toward lignin degradation and thereby reduced the organic matter mineralization [200]. A recent study had examined the effect of pyrogenic materials on soil bacteria. The study analyzed the bacteria on the phylum level. The study demonstrated that the soil type has a major role to play in shaping microbial community structure. It was observed that a few of the genera were consistent with the pyrogenic material application. They include *Mesorhizobium, Ramlibacter, Nocardioides*, and *Noviherbaspirillum* [201] .

## 4. Role of bioengineered biochar in waste recycling

### 4.1. Aerobic/Anaerobic digestion

#### 4.1.1. Composting

**Figure 2.**
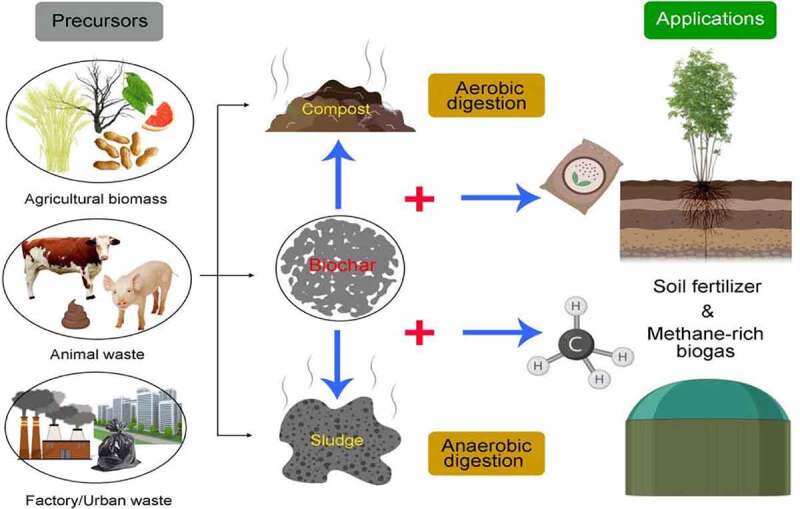
Recycling of biowaste and conversion into useful end products

Aerobic digestion (also referred to aerobic composting) is a biological process that consumes the degradable organic matter under aerobic condition, which usually accompanied by the generation of CO_2_ and NH_3_ [202]. As composting time past, harmful matters and pathogens could be degraded, while solid waste could transform into organic fertilizer. Conventional composting is a time-consuming technology that often takes place in windrows. Recently, major methods have introduced earthworms (vermicomposting) as well as biochar during the composting process, in order to shorten the time, raise the efficiency, and improve the compost characteristics (Figure 2).

Biochar as an additive during composting has been highly regarded due to its special physicochemical properties. Many studies have demonstrated that biochar addition could improve composting performance, reduce gas emissions, and adsorb heavy metals [203]. It has been reported that the high porosity of biochar is conducive to the aeration for the composting pile; hence, biochar can be used as a bulking agent. Experiment has been performed to study the utilization of biochar (derived by *Eucalyptus grandis*) for the composting [204]. The existence of biochar optimized the poultry manure compost by reducing nitrogen losses, decreasing the odor emissions, and promoting organic matter degradation. Therefore, biochar could enhance the degradation of organic matter during composting [205–207]. Results suggested that biochar adsorbs the NH_4_^+^ and H_2_S, etc., resulting in the acceleration of degradation rate of organic matter [208].

Co-composting of poultry manure and wheat straw employed biochar as an additive [209]. The results showed that biochar addition has shortened the thermophilic period of compost for about 4–5 days, which is benefit to the maturity. However, the increased temperature influenced the compost by increasing the emissions of CO_2_. Likewise, Duan et al. [210] confirmed that wheat straw derived biochar addition led to a high CO_2_ emission during the co-composting of sewage sludge. Several studies have shown opposite results, which biochar addition has decreased the CO_2_ emission [211]. In addition to CO_2_, other greenhouse gases (such as N_2_O, CH_4_, and NH_3_) are reported to be reduced due to the addition of biochar while composting. In the research of [212], although the CO_2_ emission increased, the emissions of N_2_O, CH_4_, and NH_3_ were all decreased because of the biochar addition. Also, [188]\ proved that biochar addition reduced NH_3_ emission by 58.03–65.17%, CH_4_ by 92.85–95.34% and N_2_O by 95.14–97.28%. Briefly, it is feasible to add biochar during composting for the alleviation of greenhouse gases emissions [213]. The porous structure of biochar promoted the sorption of NH_4_^+^ and NH_3_, ensured O_2_ circulation, thus improving the composting efficiency and performance. Impacts on microbial activities, contaminant eliminations, and nutrient supplements are remained to be further reviewed.

#### 4.1.2. Anaerobic digestion (AD)

AD is a conversion process that organic matter of waste was transformed into methane-rich biogas with the absence of oxygen [214,215]. Recently, biochar being applied in the AD system for bio-waste energy recycling has caused attention. Biochar is a kind of low-cost carbonaceous material with abundant functional compounds and special physicochemical performance. During the AD process, biochar could stimulate the CH_4_ production, improve the process rate, maintain the system pH and regulate microorganisms [59,216,217]. CH_4_ content reflects the AD efficiency to some extent. Corn stover biochar enhanced the production of CH_4_ in the anaerobic digestion of sewage sludge [153]. The anaerobic digestion of citrus peel waste produced 165.9 ml·g·VS^−1^ CH_4_ [218]. Comparably, the addition of wood biochar, coconut shell biochar, and rice husk biochar all increase the CH_4_ yield to 172.10, 171.30, and 186.80 ml·g·VS^−1^, respectively. The study also illustrated that biochar addition could avoid or shorten the lag phase, and accelerate the startup of AD system (Figure 2). Also, corn stover biochar increased the CH_4_ content in the produced biogas by 20.4–29.3%, and improve the digestion rate of sludge [219]. Moreover, different concentrations of biochar (1 and 10 g/L) shorten the lag phase from 10.81 d (0 g/L) to 10.48 d and 9.26 d, respectively [220]. The precursors of biochar influence the properties, resulting in different performance during AD process. Jang et al. [221] considered that the performance of wood biochar is better than wheat straw or sheep manure ones. The addition of wood biochar increased the methane yield (a 32% increase) compared with the control treatment, while wheat straw- and sheep manure-derived biochars showed no significant effect on methane generation.

**Figure 3.**
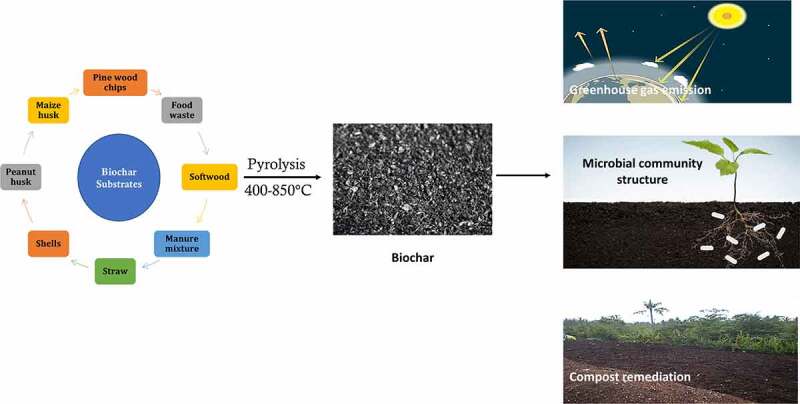
Various substrates utilized in preparation of biochar for diverse applications

pH value is a primary evaluation index for the effectiveness of the AD process, which the solution pH significantly affects the microbial functions [222]. The drop of solution pH is generally caused by volatile fatty acids (VFA) and results in the inhibition of the methane generation [217]. Fortunately, biochar has the buffer capacity that is mainly attributing to its diverse surface functional groups. [223] found that biochar addition could degrade the produced VFA and alleviated pH decrease. Besides, *Methanosarcina* is also found enriched around the biochar, suggesting the high affinity between *Methanosarcina* and biochar. It offered the adsorptive site for methanogens and stimulated the interaction between bacteria and methanogens. Magnetic biochar prevented methanogens loss and increased the methane production (11.69%) in AD process [224]. Similarly, biochar prevented the drop of pH and adsorbed methanogens, thereby shortening the lag phase and accelerating the rate of CH_4_ generation [225]. Biochar can also alter microbial community in AD process. Algae biochar at low ratios (1% and 4%, v/v) improved the enrichment of *Methanosarcina* and altered microbial community structures in AD process [226]. Additionally, biochar can improve bacteria’s growth and mediate the direct interspecies electron transfer (DIET) [227]. DIET is a common metabolism of syntrophic microorganisms [228]. The syntrophic relation between methanogens and bacteria deeply influences the efficiency of AD process [229]. Biochar as a conductive material can efficiently accelerate methanogenesis by mediating DIET. Overall, the addition of biochar in AD system plays a crucial role in CH_4_ production promoting, pH value buffering, and microbial community regulating. The precursor’s types and physicochemical properties of biochar benefited AD process differently. Nevertheless, energy recovery and cost should be considered for large-scale application (Figure 3).

### 4.2. Removal or mitigation of organic and inorganic pollutants

Nowadays, organic and inorganic (heavy metals) pollutants in environment have been mainly caused by human productive activities, including mining, electroplating, smelting, and electronic industries, chemical fertilizers, pesticide utilizations, etc.

#### 4.2.1. Adsorption of organic pollutants by biochar

Organic pollutants are extensive and complex in environment, including natural and synthetic sources. Several organic pollutant removal methods have been proposed, such as adsorption, degradation, hydrolysis, and photolysis [230]. Engineered (modified) biochar has been studied as a hotspot for its good adsorption potential. The studies of organic pollutant adsorption by biochar mainly focuses on antibiotics, phenolic compounds, dyes, volatile organic compounds, and so on. Antibiotics are developed to treat infection-related problem for human and animals. Most of them are not adsorbed or metabolized in bodies, and, therefore, have been released to the environment. Sulfonamide antibiotics have broad antibacterial activity, such as sulfamethazine (SMT), sulfamethoxazole (SMX), sulfathiazole (STZ), etc. Bamboo biomass was pyrolyzed at 380°C with H_3_PO_4_ activation to prepare biochar (named as BBC380) [231]. The maximum adsorption capacity of BBC380 showed a decrease order as STZ (237.71 mg/g) >SMX (sulfamethoxazole, 88.10 mg/g) > SMT (65.74 mg/g). Moreover, the results suggested that the surface functional groups of BBC380 may act as strong π-electron-donors and had a strong adsorption affinity for sulfonamides (π-electron acceptors). It is believed that BBC380 is very effective on sulfonamides removing. A previous study reported the preparation of steam activated tea waste-derived biochar and its utilization on the sorption of SMT [232].

The highest adsorption potential was 576.1 mg/g, above 1.5-fold better than that of the pristine biochar (342.2 mg/g). Tetracycline (TC) is also a frequently used antibiotic [233] has found that the H_3_PO_4_ modification improved the TC sorption of biochar prepared by rice straw and swine manure, especially for swine manure-derived biochar (about 25% enhancement). To date, biochar composites have raised much concern about organic pollutant removal. Bamboo was pre-treated with Graphene oxide (GO) suspension and then pyrolyzed at 600°C [234]. The SMT removal ability has been increased 1.14 times by GO-biochar compared to pristine biochar [235]. In addition, TC was adsorbed by a novel solid digestate-derived biochar-Cu NP composite with a removal efficiency of 97.8% [236].

Dyes have become a large proportion of organic pollutants in wastewater due to the development of textile industry. Methylene blue (MB) is one of the most common dyes that has been studied by many scientists. ZnCl_2_-activated biochar form tomato has been prepared and used for MB sorption [237]. Maximum MB sorption capacity has reached at 400 mg/g. Despite [238] discussed the MB sorption abilities of biochars prepared by different materials (pine wood, pig manure and cardboard), the best adsorption capacity for MB is 16.30 mg/g. In contrast, bamboo dust carbon (BDC), coconut shell carbon (CSC), groundnut shell carbon (GNSC), rice husk carbon (RHC) and straw carbon (SC) were obtained through carbonized and steam activated [239]. The MB adsorption capacities of these adsorbents are found to be of the order: BDC < GNSC < CSC < RHC < SC, with the maximum amount of 472.1 mg/g. However, biochar composites applied on dyes adsorption are still lack of studies currently.

The adsorption mechanisms of organic contaminants by biochar mainly include partitioning, pore filling, surface adsorption, hydrophobic interaction, and electrostatic interaction [240]. Several reports revealed that the adsorption of removing organic pollutants by biochar is a combination of several mechanisms. Partitioning is normally characterized by linear isotherms and non-competitive adsorption [241]. Organic pollutants are effectively complexed with the non-carbonated phase of biochar, especially with the oxygen-containing functional groups such as hydroxy and carboxy groups. [242] prepared orange peels-derived biochar and found that the isotherms of the 1-naphthol and naphthalene sorption conform to the linear relation. They also found that with the increasing of pyrolyzed temperatures (100–700°C), isotherms changed from linear to Freundliuch. This phenomenon was further elucidated as a result of multiple mechanisms including polar interaction (hydrogen bonding), π-electron interaction, pour filling, etc [243]. Because of the porous of biochar, hydrophobic-containing organic chemicals are easier to be sorbed by partitioning and hydrophobic interaction [244]. Also, a novel Fe_3_O_4_/graphene oxide/citrus peel-derived biochar reacts with the hydrophobic compounds of ciprofloxacin and sparfloxacin through the hydrophobic interaction to realize the removal of organic pollutants [245].

**Figure 4.**
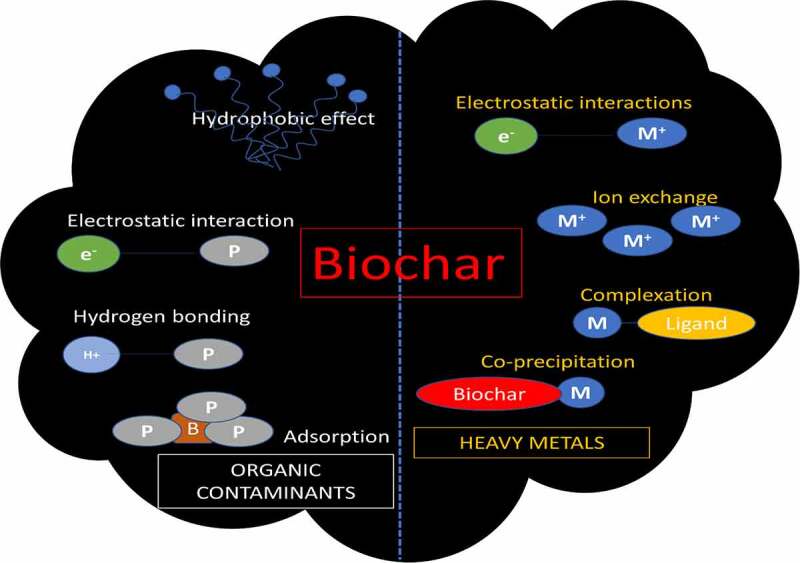
Mechanisms of biochar interactions with different pollutants. (Abbreviations: M-metal particles, M^+^-charged metal ion, e ^–^ electron, P-pollutant, B-biochar.)

Furthermore, surface adsorption occurs on the carbonated fraction of biochar, which is mainly influenced by the temperature of pyrolysis. This process is often ascribed to the physical adsorption between molecules and atoms or the chemical adsorption of molecules being attached to the surface of the adsorbent (Figure 4). Aromatic–π and cation–π interaction is a typical chemical adsorption. For instance, wood and grass pyrolyzed biochars (≥500°C) could serve as π-electron-donors and combined with phthalic acid esters, which contained aromatic-π electrons as π-acceptors, hence achieving the adsorption of phthalic acid esters by biochars [246].

#### 4.2.2. Adsorption of heavy metals by biochar

Unlike organic pollutants, heavy metals cannot be biodegraded; in contrast, heavy metals have the features of biological accumulation and magnification. Therefore, heavy metals are regarded as high bio-toxicity pollutants. Biochar, as a promising bio-adsorbent and carbon-containing material has been widely used in the removal of heavy metals in soil and water, recently. The adsorption ability of biochar for heavy metals is influenced by the nature of biochar itself on the one hand and the characteristics of metal ions on the other hand.

Surface as well as physic-chemical properties of biochar play a crucial role in heavy metal adsorption. Due to the varieties of raw materials (lignocellulosic feedstock’s, manure, and sludges, etc.) and the difference of preparation conditions (temperature, reaction time, and modification methods, etc.), the obtained biochar samples are diverse in pH value, surface area, porosity, pore distribution, and surface functional groups. Research on the heavy-metal removal of different biomass-derived biochars (Table 1) suggested that many kinds of agricultural or forestry biomasses are promising precursors of biochars. The type of feedstock’s affects biochar properties and adsorption capacity. [247] pyrolyzed rice straw (RS) and paper mill sludge (PMS) at 600°C and obtained biochars with the specific surface area of 10.9 m^2^g^−1^ and 27.2 m^2^g^−1^, respectively. It is considered that the surface area and surface functional groups make contributions to heavy-metal adsorption. However, despite the surface area is regarded as the main physical property that influences the adsorption of biochar [248], the corresponding adsorption capacity cannot be guaranteed to reach the maximum. For example, canola straw, manure pellet, sawdust and wheat straw were prepared into biochars under same conditions and activated by steam [117].

**Table 1.** Different types of raw materials and their modification

Raw materials	Preparation conditions	Modification methods	Specific surface area (m^2^g^−1^)	Heavy metals	Adsorption capacity (mg/g)	References
Corn straw	300°C, 3 h	UV-radiation	2.979	Cr(VI)	20.04	[261]
Softwood	Slow pyrolysis	170 kHz ultrasound pre-treated; NaOH activated	503.60	Cu	19.99	[262]
Hickory wood	600°C, 1 h	KMnO_4_ activated	205	Cu	14.45	[263]
Pinewood	600°C, 1 h	γ-Fe_2_O_3_ activated	193.1	As(V)	0.43	[264]
Yak manure	350°C, 2 h	H_2_O_2_ activated	6.36	Pb	169.57	[265]
*Opuntia ficus* fibres	70°C, 15 h	MnO_2_ activated	/	Cu	80.60	[266]
Canola straw	700°C, 2 h	Steam activated	106	Pb	195	[267]
Manure pellet			5.1		115	
Sawdust			356		69	
Wheat straw			316		125	
Paper mill sludge	600°C	N_2_ as the purging gas	27.2	Ni	58.48	[221]
				Cu	39.37	
				Cd	37.04	
				Pb	256.41	
Rice straw			10.9	Ni	54.95	
				Cu	19.57	
				Cd	42.74	
				Pb	133.33	
Wasted kelp	500°C, 2 h	FeCl_3_∙ 6H_2_O magnetization	0.97	Cd	23.16	[268]
				Cu	55.86	
				Zn	22.22	
Wasted hijikia			63.33	Cd	19.40	
				Cu	47.75	
				Zn	19.13	
Bamboo chips	450°C, 3 h	Eggshells pre-treated	/	Pb	261.1	[269]
Hickory chips			/		220.6	
Peanut hulls			/		103.3	
Rice straw	200°C, 3 h (Hydrothermal carbonization)	FeCl_3_ catalyst	/	Cu	4.0	[270]
				Pb	6.75	
Corn stalk	800°C, 2 h	nZVI-BC	603.4	Cu	195.1	[271]
				Pb	161.9	
				Zn	109.7	
Corn stalk	500°C, 2 h	nZVI-BC (1:1)	/	Cd	33.81	[231]
				As(III)	148.5	
Rice husk	300°C, 1 h	Impregnated with Fe^3+^	/	As(V)	0.75	[272]
				Cr(VI)	0.68	
Rice straw	450°C, 3 h	Chitosan and pyromellitic dianhydride modification	62.6	Cd	38.24	[273]
				Cu	96.11	
				Pb	13.93	

The results showed clearly that sawdust-derived biochar has the largest specific surface area, followed by wheat straw, canola straw and manure pellet. All these biochars were employed to adsorb lead (Pb) and canola straw-derived biochar has the highest maximum adsorption capacity (195 mg/g), which is about 2.8 times higher than sawdust-derived biochar (69 mg/g). Similarly, [249] compared the heavy-metal removal ability of rice husk- and dairy manure-derived biochars. Rice husk-derived biochar had a larger specific surface area than dairy manure-derived biochar (27.8 m^2^g^−1^ vs 5.61 m^2^g^−1^). The results showed that the Pb, Cu, Zn, and Cd removal capacities of dairy manure-derived biochar (above 486 mmol/kg) are much higher than those of rice husk-derived biochar (less than 140 mmol/kg). It is probably because that the dairy manure-derived biochar contained mineral components such as CO_3_^2-^ and PO_4_^3-^, which were conducive to improve the adsorption capacity.

In addition, biochar showed different adsorption capacities to different types of heavy metals. At present, biochar adsorption mechanisms for heavy metals mainly include cation exchange, electrostatic interaction, co-precipitation, surface complexation, ion reduction, and physical adsorption [250]. The heavy-metal properties such as ion radius, electronegativity value, charge density, and ion potential have an impact on the adsorption potential by biochar. [251] evaluated the effect of removing heavy metals by the orchard pruning-derived biochar and found the removal trend was Pb > Cr ≫ Cu. First of all, Pb has the highest electronegativity constant (2.33) than Cr (1.90) and Cu (1.66). Among several typical heavy metals that studied most (Pb, Cu, Zn, Cr, Cd, etc.), it is generally believed that biochar has a strong adsorption capacity for Pb. For instance, olive stone-derived biochar has a maximum adsorption capacity of 179.3 mg/g, higher than Cd (59.88 mg/g) and Cu (18.22 mg/g) [227]. Similar conclusions can be found in several studies [252–254]. Additionally, Cr has a higher charge density than Cu, suggesting the probable higher columbic attraction of the biochar. The higher adsorption may also be attributed to ionic potential and radius of the hydrated ion [255].

To maximize the removal efficiency of biochars, several new strategies have been employed to alter the biochars. [256] performed a novel biochar-supported nanoscale zero-valent iron (nZVI-BC) and found it a promising material for As(III) removal. The maximum adsorption capacity of nZVI-BCwas 148.5 mg/g, which was higher than that of nZVI (3.5 mg/g) and zeolite-supported nZVI (11.52 mg/g), previously [257,258]. A novel composite material (magnetic biochar/MgFe-layered double hydroxides) was synthesized and the maximum adsorption capacity of Pb has reached at 476.25 mg/g [259]. Overall, modified biochar adsorbs heavy metals more effectively than pristine biochar. Efforts have been made about increasing surface area, introducing abundant functional groups and modifying magnetically to improve the removal ability of heavy metals by biochar.

### 4.3. Remediation of pesticide residue

Pesticide is a broadly defined term including herbicide (control weeds), insecticide (control insects and pests) and fungicide (control fungi), etc [260]. The excessive use of pesticides can cause toxic effects on environment and human health. Biochar is considered to be soil amendment due to the unique physicochemical parameters like high surface area, abundant functional groups, and low toxicity. Studies have been performed to show that biochar can decrease pesticide pollutants in soil through adsorption. The adsorption mechanisms of biochar on pesticides are similar to those of other organic pollutants. For example, 4-chloro-2-methylphenoxy (MCPA), a herbicide, is investigated on its adsorption by wheat straw biochar [261]. The results showed that MCPA accumulation has been significantly reduced by biochar and biochar-amended soil (1.0 wt% biochar). Sorption isotherms of MCPA were fitted well to the Freundlich equation. Also, 77% of atrazine (herbicide) has been removal by the biochar converted from dairy manure [262].

The dominant sorption mechanism could be atrazine partitioning into the organic phase. [263] discussed the sorption of terbuthylazine in soil amended with biosolids and biochars. Interestingly, sawdust biochar displayed higher adsorption efficiency of terbuthylazine than the biosolids (63 times higher). A comparative study has been performed by Tatarková et al. [263] about the remediation behavior of biochar for pesticide in soil. Herbicides aminocyclopyrachlor and bentazone were almost completely adsorbed by the steam activated biochar (with higher specific surface area and lower dissolved organic carbon content) amended soils. However, the adsorption of pyraclostrobin (fungicide) did not increase by the addition of biochar. In addition to herbicide, 0.5% (w/w) *Eucalyptus* spp. woodchip biochar was added for the amendment of acetamiprid (insecticide) in red soil, paddy soil, and black soil [264]. 52.3%, 27.4%, and 11.6% of the acetamiprid has been adsorbed by biochar from red soil, paddy soil, and black soil, respectively. Early [265] believed that biochar additions to soil can reduce plant uptake of pesticides. Two types of pesticides (carbofuran and chlorpyrifos) have been amended (51% and 44%, respectively) by adding 1.0% (w/w) *Eucalyptus* sp. woodchip biochar to the soil after 35 days. [266] evaluated the maize straw biochar amendment on thiacloprid pollution. Biochar promoted the adsorption of thiacloprid and changed bacterial community of the amended soil. Overall, it has been considered that biochar can significantly reduce pesticides and contribute to the remediation for pesticide-polluted soil. Biochar precursors and their applications are depicted in Table 2.

**Table 2.** Biochar precursors and their application

Biochar precursors	Soil type	Amendment ratio	Effects	References
Maize straw	Fluvo-aquic	0.5%	Enhanced soil enzyme activities	[245]
Rice straw	Anaerobic paddy	3%	Decreased denitrifying bacteria abundance and increased iron-reducing bacteria	[246]
Peanut shell	Kaolin clay	0%, 5%, 20%	Increased water holding capacity and microorganisms’ growth	[247]
Chestnut wood	/	/	Declined β-glucosidase	[250]
Maize	Sandy soil	/	Increased available water capacity	[256]
Oilseed rape, wheat straw, miscanthus straw, mixed softwood, rice husk	/	/	Decreased the concentrations of acyl-homoserine lactone	[257]
Willow	Podzolic soil	/	Reduced bioavailability of polycyclic aromatic hydrocarbon	[259]
Forest logging residues	Karst mountainous		Altered the nutrient status and microbial community structure of karst soils	[274]
*Platanus orientalis* branches	Paddy soil	3%	Enhanced the activities of soil catalase and urease	[251]
Eucalyptus wastes branches	Experimental field	30 mg/ha	Increased the activities of enzymes β-glucosidase, acid phosphatase, arylsulfatase and urease	[244]

## 5. Role of biochar on microbial dynamics and enzymes activities

Microbial activity is one of the crucial indexes that reflects soil quality. Soil microorganisms (fungi and bacteria) play an important role in organic matter decomposition, nutrient cycling, pathogen suppression, and soil aggregation [267,268]. Biochar as a carbonous material has abundant compounds and high porous structure; hence, it changes the physicochemical properties of soil and affects the dynamics of microorganisms. Soil amendment with biochar improves microbial flora of the soil [269]. Related studies have been performed on the interactions and probable mechanisms between biochar and soil microorganisms. [270] confirmed that biochar produced *via* eucalyptus wastes (branches) increased microbial quality of the soil and the activities of enzymes β-glucosidase, acid phosphatase, arylsulfatase, and urease. However, biochar may have no effect or adverse effect on the soil microbial regulation at an improper amendment ratio. As reported by [271], 0.5% (w/w) maize biochar added into the soil could enhance soil enzyme activities. The study demonstrated that with the increasing amount of biochar amendment, the enzyme activities were suppressed. The varying additional amounts of the biochar affect not only the organic carbon and total N contents, but also the soil microbial communities after 90 days incubation. It can be speculated that the regulation of biochar on soil microbial highly depends on biochar types, dosage amounts, and soil properties [268].

The large surface area and high porosity of biochar is in favor of soil microorganisms’ growth and hosting. Therefore, precursor’s types, pyrolysis conditions (temperature and reaction time), and modification methods of biochar should be considered on the utilization of soil amendment. For example, rice straw was produced at 300, 500, and 700°C (B300/B500/B700) and the obtained biochars were employed as soil amendments on a paddy soil [272]. The surface area of biochar increased from 4.40 m^2^/g to 161 m^2^/g with the increasing temperature from 300°C to 700°C. Abundance of denitrifying bacteria, such as Bacilli, was clearly decreased by biochar B300, B500, and B700 from 16.9% to 13.6, 10.0, and 7.07%, respectively. Denitrifying bacteria could cause denitrification, which further causes the loss of soil nitrogen. Besides, the abundance of *Clostridia* increased from 33.3% (control soil) to 40.6, 48.2, and 50.6%, respectively. *Clostridia* is a kind of iron-reducing bacteria, which can compete with methanogens (produce CH_4_), thereby suppressing the emission of CH_4_. In brief, this study suggested that biochar, especially with high surface area, contributes to alleviate the emission of N_2_O and CH_4_ and amend the microorganisms’ dynamics of paddy soil. Likewise, the porosity of biochar, which increased with the surface area normally, could also improve the growth of soil microorganisms [273].

Soil enzyme activity is often connected with soil organic matter, soil physical properties, and microbial activity or biomass. [274,275] investigated how the wheat straw biocharamendment decreases soil enzyme activities in a *Torreya grandis* orchard. The results showed that activities of soil cellulase, β-fructofuranosidase, nitrate, and nitrite reductase have been significantly decreased by biochar amendment. It is probably because that biochar might bind with substrates or extracellular enzymes efficiently, thus reducing the demand of enzymes. Because of the adsorption of enzyme or substrate, the reaction rates of β-glucosidase were declined in biochar amended soil [276]. Nevertheless, the mechanism of enzyme activity impacted by biochar is not entirely understood. Several researches have proposed contradictory results. The utilization of peanut shell biochar as an additive increased glucosidase, glucuronidase, and phosphomonoesterase activity [36,277] developed *Platanus orientalis* branches biochar and modified with iron, and then applied in a paddy soil. Interestingly, pristine biochar enhanced the activities of soil catalase and urease, but the activities were suppressed by Fe-modified biochar (Table 3).

**Table 3.** Effects of various biochar synthesized from different sources

Biochar substrate used	Application target	Effect on greenhouse gas emissions	Effect on remediation	Effect on microbial community	Reference
Debarked spruce and pine trees	Soil in Stagnosol and Umbrisol field	No effect on emissions of greenhouse gases (CO_2_, N_2_O, and CH_4_).	Increased Ni and Cd in Umbrisol. Increased Ni, Fe, P, Mg, K, Al, S and Cu.	NA	[43]
Sewage sludge	Anaerobic digestion of fruit wastes	Enhanced CH_4_ production.	NA	NA	[275]
Commercial biochar derived from pinewood	Chernozems (sandy clay loam and clayey)	No effect on cumulative CH_4_-C and N_2_O-N. Cumulative CO_2_-C varied non-linearly.	NA	NA	[44]
Commercial biochar derived from pinewood	Mollisols (sandy clay loam and clayey)	Increased CO_2_ and CH_4_ flux on biochar application with manure as compared to biochar alone.	NA	NA	[45]
Straw	Rice paddy field	Increased CO_2_ uptake	NA	ND	[50]
Sugarcane	Life cycle assessment of biochar production from sugar	Lowering of greenhouse gases	NA	ND	[41]
Spruce	Temperate forest soil	Increased temperate had positive response on soil CH_4_, CO_2_ and N_2_O	NA	Decrease in overall soil microbial biomass. But promoted selection of active microbial community.	[51]
Palm	Rice field	Decreased greenhouse gas emission	NA	NA	[46]
Peanut shells	Luvisol soil	Increased CH_4_ uptake.	NA	Increased relative abundance of pmoA gene. Decreased relative abundance of nirS, nosZ and nirK genes, and ammonia oxidizing archaea.	[47]
Tree branches of *Rosa anemoniflora*	Constructed wetlands	Mitigation of N_2_O and CH_4_ fluxes.	Increased total nitrogen removal.	Increased abundance of nosZ and mcrA genes, and bacteria related to N and C transformation.	[48]
Commercial biochar derived from coconut and walnut shells	Liquid pig manure storage	Enhanced NH_3_ emission.	NA	NA	[276]
Biochar composite	Soil	N_2_O and CO_2_ emissions were inhibited.	Increased the availability of P in soil.	Abundance of *Gemmatimonas* and *Sphingomonas* were increased.	[49]
Compost	Chicken manure	Reduction in CO_2_ and ammonia.	NA	Reduction in microbial pathogens.	[277]
Soil microcosm	Fimi-Orthic Anthrosol	Reduced N_2_O emission in acidic and alkaline soils.	NA	Increased ammonia oxidizing bacteria and ammonia oxidizing archaea.	[278]
Tree branches	Constructed wetlands	Reduced greenhouse gas emission.	NA	Influenced richness and biodiversity. Increased abundance of nitrifiers and denitrifiers.	[279]
Soybean straw	Calcareous soils	NA	NA	No significant effect on tomato rhizosphere bacterial community structure.	[170]
Wheat straw	Paddy field soil (Hydragric Anthrosol)	NA	NA	Increased abundance of bacteria and fungi. Increased abundance of archaea, *Mortierella* and *Westerdykella*. Decreased phytopathogens such as *Penicillium* and *Athelia*.	[280,288–290]
Chicken manure	Poultry manure composting	NA	Removal of heavy metals.	Increased abundance of heavy metal resistant bacteria.	[281,291–294,295]
Coconut shell	Soil in pilot scale reactor	NA	Removal of heavy metals	Altered heavy metal removing bacteria population.	[282,296–300]
Modified biochar	Soil	NA	NA	Increased microbial biomass.	[283,301–305]
Crop residues	Karst soil	NA	NA	Soil microbes diversity and abundance were increased	[274]
Biochar-nanocomposite	Clayey soil	Pb and Cadmium remediation	NA	NA	[284,306–312]
Tropical Malaysian tree	Agricultural soil	Increased Cd remediation.	Suppressed nitrous oxide production precursor.	Enhanced abundance of nitrifying and denitrifying bacteria.	[148]
Wood waste biochar	Microcosm soil	Increased uptake of chiral pesticide metalaxyl	NA	Enhanced abundance of pesticide degrading bacteria.	[147]

Biochar affects the soil bulk density, aeration condition, water content, cation exchange capacity, and pH, thereby modifying soil microbial community and enzyme activity [278,279]. As biochar influences these soil physiochemical properties, the adsorption efficiency between enzymes and substrates likely depends on the characteristics (structures and chemical compounds) of amended biochar and the types of soil enzyme. There have been hypotheses about how biochar influences microbial dynamics and enzyme activities and functions as soil amendment. Direct influences mainly refer to the point that biochar provides nutrients and shelters for microorganisms [280,281]. Besides, biochar degrades soil contaminants, improves soil properties, affects adsorption of enzymes, interrupts the communications between microbial cells, and ultimately affects the microbial activity [282–286,287]. Therefore, effects of biochar as soil amendment are needed to be further investigated and the underlying mechanisms are still remained to be clarified.

## 6. Conclusions and future prospects

Biochar, a by-product of engineered waste biomass, has a lot of potential for reducing environmental impact, combating climate change, and establishing a circular economy. Biochar is an economically viable waste treatment material because of its reusability and quick availability, regardless of the season. Collaborative research activities focused on producers, consumers, government is essential for the fruitful promotion of biochar-based circular economy technologies. Overall, it was discovered that the biowaste elements, in combination with the engineered process parameters, play a pivotal role in the yield and composition of the biochars produced. As a result, it is critical to perform additional studies focused on discovering production methodologies and biochar qualities for circular economy-based sustainable production.

- A unified standard is required for the biochar industry’s healthy and long-term development.
- Intense research on merits and limitations of biochar for various soil applications to be carried out to develop an economically viable strategy for its commercialization on industrial scale.
- Impact of biochar properties on various applications to be explored in depth.
- Research and development activities to be more focused on developing eco-friendly and economically viable strategies for the production and applications of biochar.
- There is an urgent need to fill knowledge gaps in areas like long-term carbon sequestration, pollution release, and the influence of biochar on ecological systems.
- Fruitful collaboration between industry, testing centers, and government is essential for the creation of the biochar-based circular economy market.
- In response to changing markets, more attention should be directed at biochar research, innovation, and creation.

## Highlights

- Porosity and hydrophobicity of biochar determines the adsorption efficiency.
- Bioengineered biochar is found to be more efficient in bioremediation.
- Diverse applications of biochar are discussed.

## References

[1] Lag-Brotons AJ, Velenturf APM, Crane R, et al. Editorial: resource recovery from waste. Editorial. 2020;8:35.

[2] Awasthi MK, Duan Y, Awasthi SK, et al. Influence of bamboo biochar on mitigating greenhouse gas emissions and nitrogen loss during poultry manure composting. Bioresour Technol. 2020;303:122952. 32050126 10.1016/j.biortech.2020.122952

[3] Jain A, Sarsaiya S, Awasthi MK, et al. Bioenergy and bio-products from bio-waste and its associated modern circular economy: current research trends, challenges, and future outlooks. Fuel. 2022;307:121859.

[4] Murray A, Skene K, Haynes K. The circular economy: an interdisciplinary exploration of the concept and application in a global context. J Bus Ethics. 2017;140(3):369–380.

[5] García-Barragán JF, Eyckmans J, Defining RS. Measuring the circular economy: a mathematical approach. Ecol Econ. 2019;157:369–372.

[6] Velenturf APM, Archer SA, Gomes HI, et al. Circular economy and the matter of integrated resources. Sci Total Environ. 2019;689:963–969. 31280177 10.1016/j.scitotenv.2019.06.449

[7] Hu Q, Jung J, Chen D, et al. Biochar industry to circular economy. Sci Total Environ. 2021;757:143820. 33248779 10.1016/j.scitotenv.2020.143820

[8] Awasthi MK, Pandey AK, Khan J, et al. Evaluation of thermophilic fungal consortium for organic municipal solid waste composting. Bioresour Technol. 2014;168:214–221. 24507579 10.1016/j.biortech.2014.01.048

[9] Awasthi MK, Pandey AK, Bundela PS, et al. Co-composting of organic fraction of municipal solid waste mixed with different bulking waste: Characterization of physicochemical parameters and microbial enzymatic dynamic. Bioresour Technol. 2015;182:200–207. 25698412 10.1016/j.biortech.2015.01.104

[10] Prajapati P, Varjani S, Singhania RR, et al. Critical review on technological advancements for effective waste management of municipal solid waste-Updates and way forward. Environ Technol Innov. 2021;23:101749.

[11] Awasthi MK, Duan Y, Awasthi SK, et al. Emerging applications of biochar: Improving pig manure composting and attenuation of heavy metal mobility in mature compost. J Hazard Mater. 2020;389:122116. 31972527 10.1016/j.jhazmat.2020.122116

[12] Awasthi MK, Wang Q, Wang M, et al. In-Vessel co-composting of food waste employing enriched bacterial consortium. Food Technol Biotechnol. 2018;56(1):83–89. DOI:10.17113/ftb.56.01.18.5439. 29796000 PMC5956273

[13] Awasthi MK, Wang Q, Huang H, et al. Effect of biochar amendment on greenhouse gas emission and bio-availability of heavy metals during sewage sludge co-composting. J Clean Prod. 2016;135:829–835.

[14] Awasthi MK, Sarsaiya S, Wainaina S, et al. Techno-economics and life-cycle assessment of biological and thermochemical treatment of bio-waste. Renew Sust Energ Rev. 2021;144:110837.

[15] Duan Y, Pandey A, Zhang Z, et al. Organic solid waste biorefinery: sustainable strategy for emerging circular bioeconomy in China. Ind Crops Prod. 2020;153:112568.

[16] Liu HM, Qin SY, Sirohi R, et al. Sustainable blueberry waste recycling towards biorefinery strategy and circular bioeconomy: a review. Bioresour Technol. 2021;332:125181. 33888357 10.1016/j.biortech.2021.125181

[17] Duan W, Oleszczuk P, Pan B, et al. Environmental behavior of engineered biochars and their aging processes in soil. Biochar. 2019;1(4):339–351. DOI:10.1007/s42773-019-00030-5.

[18] Arora S, Jung J, Liu M, et al. Gasification biochar from horticultural waste: an exemplar of the circular economy in Singapore. Sci Total Environ. 2021;781:146573. 33798876 10.1016/j.scitotenv.2021.146573

[19] Lee JTE, Ok YS, Song S, et al. Biochar utilisation in the anaerobic digestion of food waste for the creation of a circular economy via biogas upgrading and digestate treatment. Bioresour Technol. 2021;333:125190. 33915456 10.1016/j.biortech.2021.125190

[20] Jindo K, Audette Y, Higashikawa FS, et al. Role of biochar in promoting circular economy in the agriculture sector. Part 1: a review of the biochar roles in soil N, P and K cycles. Chem Biol Technol Agric. 2020a;7(1):15. DOI:10.1186/s40538-020-00182-8.

[21] Jindo K, Sánchez-Monedero MA, Mastrolonardo G, et al. Role of biochar in promoting circular economy in the agriculture sector. Part 2: a review of the biochar roles in growing media, composting and as soil amendment. Chem Biol Technol Agric. 2020b;7(1):16. DOI:10.1186/s40538-020-00179-3.

[22] Awasthi MK, Wang MJ, Pandey A, et al. Heterogeneity of zeolite combined with biochar properties as a function of sewage sludge composting and production of nutrient-rich compost. Waste Manage. 2017;68:760–773. 10.1016/j.wasman.2017.06.008 28623022

[23] Panahi HKS, Dehhaghi M, Ok YS, et al. A comprehensive review of engineered biochar: production, characteristics, and environmental applications. J Clean Prod. 2020;270:122462.

[24] Awasthi MK, Chen H, Wang Q, et al. Succession of bacteria diversity in the poultry manure composted mixed with clay: studies upon its dynamics and associations with physicochemical and gaseous parameters. Bioresour Technol. 2018;267:618–625. 30056372 10.1016/j.biortech.2018.07.094

[25] Dehhaghi M, Tabatabaei M, Aghbashlo M, et al. A state-of-the-art review on the application of nanomaterials for enhancing biogas production. J Environ Manage. 2019;251:109597. 31563049 10.1016/j.jenvman.2019.109597

[26] Tu C, Wei J, Guan F, et al. Biochar and bacteria inoculated biochar enhanced Cd and Cu immobilization and enzymatic activity in a polluted soil. Environ Int. 2020;137:105576. 32070805 10.1016/j.envint.2020.105576

[27] Anae J, Ahmad N, Kumar V, et al. Recent advances in biochar engineering for soil contaminated with complex chemical mixtures: remediation strategies and future perspectives. Sci Total Environ. 2021;767:144351. 33453509 10.1016/j.scitotenv.2020.144351

[28] Frankel ML, Bhuiyan TI, Veksha A, et al. Removal and biodegradation of naphthenic acids by biochar and attached environmental biofilms in the presence of co-contaminating metals. Bioresour Technol. 2016;216:352–361. 27259191 10.1016/j.biortech.2016.05.084

[29] Dalahmeh S, Ahrens L, Gros M, et al. Potential of biochar filters for onsite sewage treatment: adsorption and biological degradation of pharmaceuticals in laboratory filters with active, inactive and no biofilm. Sci Total Environ. 2018;612:192–201. 28850838 10.1016/j.scitotenv.2017.08.178

[30] Yao Y, Zhang Y, Gao B, et al. Removal of sulfamethoxazole (SMX) and sulfapyridine (SPY) from aqueous solutions by biochars derived from anaerobically digested bagasse. Environ Sci Pollut Res. 2018;25(26):25659–25667. DOI:10.1007/s11356-017-8849-0. 28353104

[31] Shirzad M, Kazemi Shariat PH, Bb D, et al. A comprehensive review on electricity generation and GHG emission reduction potentials through anaerobic digestion of agricultural and livestock/slaughterhouse wastes in Iran. Renew. Sust Energ Rev. 2019;111:571–594.

[32] Wainaina S, Lukitawesa AMK, Kumar Awasthi M, et al. Bioengineering of anaerobic digestion for volatile fatty acids, hydrogen or methane production: a critical review. Bioengineered. 2019;10(1):437–458. DOI:10.1080/21655979.2019.1673937. 31570035 PMC6802927

[33] Jeon J, Kim H-I, Park JH, et al. Evaluation of thermal properties and acetaldehyde adsorption performance of sustainable composites using waste wood and biochar. Environ Res. 2021;196:110910. 33639144 10.1016/j.envres.2021.110910

[34] Haris M, Hamid Y, Usman M, et al. Crop-residues derived biochar: synthesis, properties, characterization and application for the removal of trace elements in soils. J Hazard Mater. 2021;416:126212.

[35] Akdeniz N. A systematic review of biochar use in animal waste composting. Waste Manage. 2019;88:291–300. 10.1016/j.wasman.2019.03.054 31079642

[36] Dominchin MF, Verdenelli RA, Berger MG, et al. Impact of N-fertilization and peanut shell biochar on soil microbial community structure and enzyme activities in a typic Haplustoll under different management practices. Eur J Soil Biol. 2021;104:103298.

[37] Mona S, Malyan SK, Saini N, et al. Towards sustainable agriculture with carbon sequestration, and greenhouse gas mitigation using algal biochar. Chemosphere. 2021;275:129856. 33636519 10.1016/j.chemosphere.2021.129856

[38] Ghanim B, Murnane JG, O’Donoghue L, et al. Removal of vanadium from aqueous solution using a red mud modified saw dust biochar. J Water Process Eng. 2020;33:101076.

[39] Azadi N, Raiesi F. Sugarcane bagasse biochar modulates metal and salinity stresses on microbial functions and enzyme activities in saline co-contaminated soils. Appl Soil Ecol. 2021;167:104043.

[40] Khalil U, Bilal Shakoor M, Ali S, et al. Adsorption-reduction performance of tea waste and rice husk biochars for Cr (VI) elimination from wastewater. J Saudi Chem Soc. 2020;24(11):799–810. DOI:10.1016/j.jscs.2020.07.001.

[41] Bhuvaneshwari S, Hettiarachchi H, Meegoda JN. Crop residue burning in India: policy challenges and potential solutions. Int J Environ. 2019;16(5):832. 10.3390/ijerph16050832 PMC6427124 30866483

[42] Awasthi MK, Duan Y, Liu T, et al. Relevance of biochar to influence the bacterial succession during pig manure composting. Bioresour Technol. 2020;304:122962. 32066092 10.1016/j.biortech.2020.122962

[43] El-Naggar A, El-Naggar AH, Shaheen SM, et al. Biochar composition-dependent impacts on soil nutrient release, carbon mineralization, and potential environmental risk: a review. J Environ Manage. 2019;241:458–467. 31027831 10.1016/j.jenvman.2019.02.044

[44] Bilgili AV, Aydemir S, Altun O, et al. The effects of biochars produced from the residues of locally grown crops on soil quality variables and indexes. Geoderma. 2019;345:123–133.

[45] Su X, Wang Y, He Q, et al. Biochar remediates denitrification process and N_2_O emission in pesticide chlorothalonil-polluted soil: Role of electron transport chain. Chem Eng J. 2019;370:587–594.

[46] Aggangan NS, Cortes RB, Opulencia JG, et al. Effects of mycorrhizal fungi and bamboo biochar on the rhizosphere bacterial population and nutrient uptake of cacao (*Theobroma cacao* L.) Seedlings. Philipp J Crop Sci. 2019;44(1):1–9.

[47] Solaiman ZM, Abbott LK, Murphy DV. Biochar phosphorus concentration dictates mycorrhizal colonisation, plant growth and soil phosphorus cycling. Sci Rep. 2019;9(1):1–11. 30911114 10.1038/s41598-019-41671-7 PMC6433866

[48] Zhou Y, Qin S, Verma S, et al. Production and beneficial impact of biochar for environmental application: a comprehensive review. Bioresour Technol. 2021;337:125451. 34186328 10.1016/j.biortech.2021.125451

[49] Yu J, Deem LM, Crow SE, et al. Comparative metagenomics reveals enhanced nutrient cycling potential after 2 years of biochar amendment in a tropical oxisol. Appl Environ Microbiol. 2019;85(11):02957–02918. DOI:10.1128/AEM.02957-18. PMC6532032 30952661

[50] Liu Q, Liu B, Zhang Y, et al. Biochar application as a tool to decrease soil nitrogen losses (NH_3_ volatilization, N_2_O emissions, and N leaching) from croplands: Options and mitigation strength in a global perspective. Glob Chang Biol. 2019;25(6):2077–2093. DOI:10.1111/gcb.14613. 30844112

[51] Gautam RK, Mishra RK, Chaturvedi P, et al. Biochar for remediation of agrochemicals and synthetic organic dyes from environmental samples: A review. Chemosphere. 2021;272:129917. 10.1016/j.chemosphere.2021.129917 35534974

[52] Chen H, Awasthi SK, Liu T, et al. Effects of microbial culture and chicken manure biochar on compost maturity and greenhouse gas emissions during chicken manure composting. J Hazard Mater. 2020;389:121908. 31879100 10.1016/j.jhazmat.2019.121908

[53] Awasthi SK, Duan Y, Liu T, et al. Sequential presence of heavy metal resistant fungal communities influenced by biochar amendment in the poultry manure composting process. J Clean Prod. 2021;291:125947.

[54] Ye L, Camps‐Arbestain M, Shen Q, et al. Biochar effects on crop yields with and without fertilizer: a meta‐analysis of field studies using separate controls. Soil Use Manag. 2020;36(1):2–18. DOI:10.1111/sum.12546.

[55] Dhyani V, Awasthi MK, Wang Q, et al. Effect of composting on the thermal decomposition behavior and kinetic parameters of pig manure-derived solid waste. Bioresour Technol. 2018;252:59–65. 29306130 10.1016/j.biortech.2017.12.083

[56] Liu T, Awasthi SK, Duan Y, et al. Current status of global warming potential reduction by cleaner composting. Energy Environ. 2019;32(6):1–27.

[57] Hoegh-Guldberg O, Jacob D, Taylor M, et al. The human imperative of stabilizing global climate change at 1.5 C. Science. 2019;365(6459). DOI:10.1126/science.aaw6974. 31604209

[58] Lefebvre D, Williams A, Kirk GJD, et al. An anticipatory life cycle assessment of the use of biochar from sugarcane residues as a greenhouse gas removal technology. J Clean Prod. 2021;312:127764.

[59] Kumar M, Dutta S, You S, et al. A critical review on biochar for enhancing biogas production from anaerobic digestion of food waste and sludge. J Clean. 2021;305:127143.

[60] Duan Y, Awasthi SK, Liu T, et al. Evaluation of integrated biochar with bacterial consortium on gaseous emissions mitigation and nutrients sequestration during pig manure composting. Bioresour Technol. 2019;291:121880. 31374415 10.1016/j.biortech.2019.121880

[61] Awasthi MK, Awasthi SK, Wang Q, et al. Influence of biochar on volatile fatty acids accumulation and microbial community succession during biosolids composting. Bioresour Technol. 2018;251:158–164. 29274855 10.1016/j.biortech.2017.12.037

[62] Awasthi MK, Liu T, Awasthi SK, et al. Manure pretreatments with black soldier fly Hermetia illucens L. (Diptera: Stratiomyidae): A study to reduce pathogen content. Sci Total Environ. 2020;737:139842. 32526587 10.1016/j.scitotenv.2020.139842

[63] Awasthi MK, Wang M, Chen H, et al. Heterogeneity of biochar amendment to improve the carbon and nitrogen sequestration through reduce the greenhouse gases emissions during sewage sludge composting. Bioresour Technol. 2017;224:428–438. 27843087 10.1016/j.biortech.2016.11.014

[64] Kalu S, Simojoki A, Karhu K, et al. Long-term effects of softwood biochar on soil physical properties, greenhouse gas emissions and crop nutrient uptake in two contrasting boreal soils. Agric Ecosyst Environ. 2021;316:107454.

[65] Romero CM, Hao X, Li C, et al. Nutrient retention, availability and greenhouse gas emissions from biochar-fertilized chernozems. Catena. 2021;198:105046.

[66] Romero CM, Li C, Owens J, et al. Nutrient cycling and greenhouse gas emissions from soil amended with biochar-manure mixtures. Pedosphere. 2021;31(2):289–302. DOI:10.1016/S1002-0160(20)60071-6.

[67] Shin J, Park D, Hong S, et al. Influence of activated biochar pellet fertilizer application on greenhouse gas emissions and carbon sequestration in rice (*Oryza sativa* L.) production. Environ Pollut. 2021;285:117457. 34380210 10.1016/j.envpol.2021.117457

[68] He T, Yun F, Liu T, et al. Differentiated mechanisms of biochar-and straw-induced greenhouse gas emissions in tobacco fields. Appl Soil Ecol. 2021;166:103996.

[69] Ji B, Chen J, Li W, et al. Greenhouse gas emissions from constructed wetlands are mitigated by biochar substrates and distinctly affected by tidal flow and intermittent aeration modes. Environ Pollut. 2021;271:116328. 33360581 10.1016/j.envpol.2020.116328

[70] Liu Z, Tang J, Ren X, et al. Effects of phosphorus modified nzvi-biochar composite on emission of greenhouse gases and changes of microbial community in soil. Environ Pollut. 2021;274:116483. 33508717 10.1016/j.envpol.2021.116483

[71] Cao Y, Shan Y, Wu P, et al. Mitigating the global warming potential of rice paddy fields by straw and straw-derived biochar amendments. Geoderma. 2021;396:115081.

[72] Cui J, Glatzel S, Bruckman VJ, et al. Long-term effects of biochar application on greenhouse gas production and microbial community in temperate forest soils under increasing temperature. Sci Total Environ. 2021;767:145021. 33636794 10.1016/j.scitotenv.2021.145021

[73] Awasthi MK, Wang Q, Chen H, et al. In-vessel co-composting of biosolid: focusing on mitigation of greenhouse gases emissions and nutrients conservation. Renew Energ. 2018;129:814–823.

[74] Xue S, Zhou L, Zhong M, et al. Bacterial agents affected bacterial community structure to mitigate greenhouse gas emissions during sewage sludge composting. Bioresour Technol. 2021;337:125397. 34139563 10.1016/j.biortech.2021.125397

[75] Borchard N, Schirrmann M, Cayuela ML, et al. Biochar, soil and land-use interactions that reduce nitrate leaching and N_2_O emissions: a meta-analysis. Sci Total Environ. 2019;651:2354–2364. 30336425 10.1016/j.scitotenv.2018.10.060

[76] Zhang Q, Xiao J, Xue J, et al. Quantifying the effects of biochar application on greenhouse gas emissions from agricultural soils: a global meta-analysis. Sustainability. 2020;12(8):3436. DOI:10.3390/su12083436.

[77] Cao Y, Wang X, Bai Z, et al. Mitigation of ammonia, nitrous oxide and methane emissions during solid waste composting with different additives: a meta-analysis. J Clean Prod. 2019;235:626–635.

[78] Pereira JL, Figueiredo V, Pinto AF, et al. Effects of biochar and clinoptilolite on composition and gaseous emissions during the storage of separated liquid fraction of pig slurry. Appl Sci. 2020;10(16):5652. DOI:10.3390/app10165652.

[79] Wu Z, Zhang Q, Zhang X, et al. Biochar-enriched soil mitigated N_2_O and NO emissions similarly as fresh biochar for wheat production. Sci Total Environ. 2020;701:134943. 31731203 10.1016/j.scitotenv.2019.134943

[80] Wang H, Shen M, Hui D, et al. Straw incorporation influences soil organic carbon sequestration, greenhouse gas emission, and crop yields in a Chinese rice (*Oryza sativa* L.) –wheat (*Triticum aestivum* L.) cropping system. Soil Tillage Res. 2019;195:104377.

[81] Huang M, Li Z, Luo N, et al. Application potential of biochar in environment: Insight from degradation of biochar-derived DOM and complexation of DOM with heavy metals. Sci Total Environ. 2019;646:220–228. 30053666 10.1016/j.scitotenv.2018.07.282

[82] Wu H, Fan J, Zhang J, et al. Large-scale multi-stage constructed wetlands for secondary effluents treatment in northern China: carbon dynamics. Environ Pollut. 2018;233:933–942. 29029835 10.1016/j.envpol.2017.09.048

[83] Guo F, Zhang J, Yang X, et al. Impact of biochar on greenhouse gas emissions from constructed wetlands under various influent chemical oxygen demands to nitrogen ratios. Bioresour Technol. 2020;303:122908. 32028219 10.1016/j.biortech.2020.122908

[84] Saeed T, Miah MJ, Khan T, et al. Pollutant removal employing tidal flow constructed wetlands: media and feeding strategies. Chem Eng J. 2020;382:122874.

[85] Li J, Fan J, Liu D, et al. Enhanced nitrogen removal in biochar-added surface flow constructed wetlands: dealing with seasonal variation in the north China. Environ Sci Pollut Res. 2019;26(4):3675–3684. DOI:10.1007/s11356-018-3895-9. 30535737

[86] Chen X, Zhu H, Banuelos G, et al. Biochar reduces nitrous oxide but increases methane emissions in batch wetland mesocosms. Chem Eng J. 2020;392:124842.

[87] Zhang K, Liu Y, Chen Q, et al. Effect of submerged plant species on CH_4_ flux and methanogenic community dynamics in a full-scale constructed wetland. Ecol Eng. 2018;115:96–104.

[88] Zhuang -L-L, Yang T, Zhang J, et al. The configuration, purification effect and mechanism of intensified constructed wetland for wastewater treatment from the aspect of nitrogen removal: a review. Bioresour Technol. 2019;293:122086. 31495460 10.1016/j.biortech.2019.122086

[89] Huang T, Liu W, Zhang Y, et al. A stable simultaneous anammox, denitrifying anaerobic methane oxidation and denitrification process in integrated vertical constructed wetlands for slightly polluted wastewater. Environ Pollut. 2020;262:114363. 32443207 10.1016/j.envpol.2020.114363

[90] Iqbal A, Shang Z, Rehman MLU, et al. Pattern of microbial community composition and functional gene repertoire associated with methane emission from Zoige wetlands, China—A review. Sci Total Environ. 2019;694:133675. 31756831 10.1016/j.scitotenv.2019.133675

[91] Pishgar R, Dominic JA, Sheng Z, et al. Denitrification performance and microbial versatility in response to different selection pressures. Bioresour Technol. 2019;281:72–83. 30798089 10.1016/j.biortech.2019.02.061

[92] Liang Y, Wang Q, Huang L, et al. Insight into the mechanisms of biochar addition on pollutant removal enhancement and nitrous oxide emission reduction in subsurface flow constructed wetlands: microbial community structure, functional genes and enzyme activity. Bioresour Technol. 2020;307:123249. 32244072 10.1016/j.biortech.2020.123249

[93] Yoo G, Lee YO, Won TJ, et al. Variable effects of biochar application to soils on nitrification-mediated N_2_O emissions. Sci Total Environ. 2018;626:603–611. 29358139 10.1016/j.scitotenv.2018.01.098

[94] Fan C, Duan P, Zhang X, et al. Mechanisms underlying the mitigation of both N_2_O and NO emissions with field-aged biochar in an Anthrosol. Geoderma. 2020;364:114178.

[95] Duan P, Song Y, Li S, et al. Responses of N_2_O production pathways and related functional microbes to temperature across greenhouse vegetable field soils. Geoderma. 2019;355:113904.

[96] Jiang Y, Qian H, Huang S, et al. Acclimation of methane emissions from rice paddy fields to straw addition. Sci Adv. 2019;5(1):eaau9038. DOI:10.1126/sciadv.aau9038. 30746466 PMC6357747

[97] Evans PN, Boyd JA, Leu AO, et al. An evolving view of methane metabolism in the Archaea. Nat Rev Microbiol. 2019;17(4):219–232. DOI:10.1038/s41579-018-0136-7. 30664670

[98] Lv X, Wang Z, Ma L, et al. Crop residue incorporation combined with potassium fertilizer increased cotton canopy apparent photosynthesis and seed cotton yield in barley-cotton rotation system. Arch Agron Soil Sci. 2021;67(3):300–312. DOI:10.1080/03650340.2020.1723160.

[99] Zhang Q, Song Y, Wu Z, et al. Effects of six-year biochar amendment on soil aggregation, crop growth, and nitrogen and phosphorus use efficiencies in a rice-wheat rotation. J Clean Prod. 2020;242:118435.

[100] Awasthi SK, Duan Y, Liu T, et al. Can biochar regulate the fate of heavy metals (Cu and Zn) resistant bacteria community during the poultry manure composting? J. Hazard Mater. 2021;406:124593. 10.1016/j.jhazmat.2020.124593 33316669

[101] Peng Z, Liu X, Chen H, et al. Characterization of ultraviolet-modified biochar from different feedstocks for enhanced removal of hexavalent chromium from water. Water Sci Technol. 2019;79(9):1705–1716. DOI:10.2166/wst.2019.170. 31241476

[102] Liu P, Li H, Liu X, et al. Preparation of magnetic biochar obtained from one-step pyrolysis of *Salix mongolica* and investigation into adsorption behavior of sulfadimidine sodium and norfloxacin in aqueous solution. J Dispers Sci Technol. 2020;41(2):214–226. DOI:10.1080/01932691.2018.1562354.

[103] Gielnik A, Pechaud Y, Huguenot D, et al. Bacterial seeding potential of digestate in bioremediation of diesel contaminated soil. Int Biodeterior Biodegradation. 2019;143:104715.

[104] Gielnik A, Pechaud Y, Huguenot D, et al. Effect of digestate application on microbial respiration and bacterial communities’ diversity during bioremediation of weathered petroleum hydrocarbons contaminated soils. Sci Total Environ. 2019;670:271–281. 30903900 10.1016/j.scitotenv.2019.03.176

[105] Sajjadi B, Zubatiuk T, Leszczynska D, et al. Chemical activation of biochar for energy and environmental applications: a comprehensive review. Rev Chem Eng. 2019;35(7):777–815. DOI:10.1515/revce-2018-0003.

[106] Sajjadi B, Chen W-Y, Egiebor NO. A comprehensive review on physical activation of biochar for energy and environmental applications. Rev Chem Eng. 2019;35(6):735–776.

[107] Inyang M, Gao B, Pullammanappallil P, et al. Biochar from anaerobically digested sugarcane bagasse. Bioresour Technol. 2010;101(22):8868–8872. DOI:10.1016/j.biortech.2010.06.088. 20634061

[108] Gielnik A, Pechaud Y, Huguenot D, et al. Potential use of waste-to-bioenergy by-products in bioremediation of Total Petroleum Hydrocarbons (TPH)-Contaminated Soils. In: van Hullebusch E., Huguenot D., Pechaud Y., Simonnot MO., Colombano S. (eds) Environmental Soil Remediation and Rehabilitation. Applied Environmental Science and Engineering for a Sustainable Future. Cham: Springer; 2020. p. 239–282. 10.1007/978-3-030-40348-5_5

[109] Gielnik A, Pechaud Y, Huguenot D, et al. Functional potential of sewage sludge digestate microbes to degrade aliphatic hydrocarbons during bioremediation of a petroleum hydrocarbons contaminated soil. J Environ Manage. 2021;280:111648. 33213993 10.1016/j.jenvman.2020.111648

[110] Wang J, Wang S. Preparation, modification and environmental application of biochar: a review. J Clean Prod. 2019;227:1002–1022.

[111] Maguyon-Detras MC, Migo MVP, Van Hung N, et al. Thermochemical conversion of rice straw. In: Gummert M., Hung N., Chivenge P., Douthwaite B. (eds) Sustainable Rice Straw Management. 2020. p. 43–64. Cham: Springer. 10.1007/978-3-030-32373-8_4

[112] Wang L, Ok YS, Tsang DC, et al. New trends in biochar pyrolysis and modification strategies: feedstock, pyrolysis conditions, sustainability concerns and implications for soil amendment. Soil Use Manag. 2020;36(3):358–386. DOI:10.1111/sum.12592.

[113] Wang X, Guo Z, Hu Z, et al. Recent advances in biochar application for water and wastewater treatment: a review. Peer J. 2020;8:9164. 10.7717/peerj.9164 PMC7243815 32477836

[114] Tomczyk A, Sokołowska Z, Boguta P. Biochar physicochemical properties: pyrolysis temperature and feedstock kind effects. Rev Environ Sci Biotechnol. 2020;19(1):191–215.

[115] Paunovic O, Pap S, Maletic S, et al. Ionisable emerging pharmaceutical adsorption onto microwave functionalised biochar derived from novel lignocellulosic waste biomass. J Colloid Interface Sci. 2019;547:350–360. 30974250 10.1016/j.jcis.2019.04.011

[116] Braghiroli FL, Bouafif H, Koubaa A. Enhanced SO_2_ adsorption and desorption on chemically and physically activated biochar made from wood residues. Ind Crops Prod. 2019;138:111456.

[117] Kwak JH, Islam MS, Wang S, et al. Biochar properties and lead (II) adsorption capacity depend on feedstock type, pyrolysis temperature, and steam activation. Chemosphere. 2019;231:393–404. 31146131 10.1016/j.chemosphere.2019.05.128

[118] Pandey D, Daverey A, Arunachalam K. Biochar: Production, properties and emerging role as a support for enzyme immobilization. Journal of Cleaner Production. 2020;255:120267.

[119] Leng L, Huang H. An overview of the effect of pyrolysis process parameters on biochar stability. Bioresour Technol. 2018;270:627–642. 30220436 10.1016/j.biortech.2018.09.030

[120] Yang X, Tsibart A, Nam H, et al. Effect of gasification biochar application on soil quality: Trace metal behavior, microbial community, and soil dissolved organic matter. J Hazard Mater. 2019;365:684–694. 30472454 10.1016/j.jhazmat.2018.11.042

[121] Awasthi MK, Ravindran B, Sarsaiya S, et al. Metagenomics for taxonomy profiling: tools and approaches. Bioengineered. 2020;11(1):356–374. DOI:10.1080/21655979.2020.1736238. 32149573 PMC7161568

[122] Varma AK, Thakur LS, Shankar R, et al. Pyrolysis of wood sawdust: Effects of process parameters on products yield and characterization of products. Waste Manage. 2019;89:224–235. 10.1016/j.wasman.2019.04.016 31079735

[123] Yang F, Zhang S, Sun Y, et al. A novel electrochemical modification combined with one-step pyrolysis for preparation of sustainable thorn-like iron-based biochar composites. Bioresour Technol. 2019;274:379–385. 30544043 10.1016/j.biortech.2018.10.042

[124] Nie C, Yang X, Niazi NK, et al. Impact of sugarcane bagasse-derived biochar on heavy metal availability and microbial activity: a field study. Chemosphere. 2018;200:274–282. 29494908 10.1016/j.chemosphere.2018.02.134

[125] Cipullo S, Negrin I, Claveau L, et al. Linking bioavailability and toxicity changes of complex chemicals mixture to support decision making for remediation endpoint of contaminated soils. Sci Total Environ. 2019;650:2150–2163. 30290356 10.1016/j.scitotenv.2018.09.339

[126] Oleszczuk P, Rakowska M, Bucheli TD, et al. Combined effects of plant cultivation and sorbing carbon amendments on freely dissolved PAHs in contaminated soil. Environ Sci Technol. 2019;53(9):4860–4868. DOI:10.1021/acs.est.8b06265. 30920807

[127] Chen X, He H-Z, Chen G-K, et al. Effects of biochar and crop straws on the bioavailability of cadmium in contaminated soil. Sci Rep. 2020;10(1):1–12. 32533061 10.1038/s41598-020-65631-8 PMC7293325

[128] Alaboudi KA, Ahmed B, Brodie G. Effect of biochar on Pb, Cd and Cr availability and maize growth in artificial contaminated soil. Ann Agric Sci. 2019;64(1):95–102.

[129] Crisler GB, Burk GA, Simmons P, et al. Lead removal using biochars obtained from slow pyrolysis of dry and water-soaked pecan shell biomass. Sep Sci Technol. 2020;55(11):1947–1956. DOI:10.1080/01496395.2019.1617740.

[130] Igalavithana AD, Kwon EE, Vithanage M, et al. Soil lead immobilization by biochars in short-term laboratory incubation studies. Environ Int. 2019;127:190–198. 30925262 10.1016/j.envint.2019.03.031

[131] Piscitelli L, Malerba AD, Mezzapesa GN, et al. Potential microbial remediation of pyrene polluted soil: the role of biochar. Soil Res. 2019;57(8):807–813. DOI:10.1071/SR19075.

[132] Zhang G, He L, Guo X, et al. Mechanism of biochar as a biostimulation strategy to remove polycyclic aromatic hydrocarbons from heavily contaminated soil in a coking plant. Geoderma. 2020;375:114497.

[133] Zhang M, Yin Q, Ji X, et al. High and fast adsorption of Cd (II) and Pb (II) ions from aqueous solutions by a waste biomass based hydrogel. Sci Rep. 2020;10(1):1–13. 32094399 10.1038/s41598-020-60160-w PMC7040188

[134] Li X, Li Y, Zhang X, et al. Long-term effect of biochar amendment on the biodegradation of petroleum hydrocarbons in soil microbial fuel cells. Sci Total Environ. 2019;651:796–806. 30253361 10.1016/j.scitotenv.2018.09.098

[135] Li X, Song Y, Wang F, et al. Combined effects of maize straw biochar and oxalic acid on the dissipation of polycyclic aromatic hydrocarbons and microbial community structures in soil: a mechanistic study. J Hazard Mater. 2019;364:325–331. 30384242 10.1016/j.jhazmat.2018.10.041

[136] Deng L, Liu Y, Wang W. Utilization of digestate. In: Deng, L., Liu, Y., Wang, W. (eds) Biogas Technology. Singapore: Springer; 2020. p. 319–363. 10.1007/978-981-15-4940-3_9

[137] Logan M, Visvanathan C. Management strategies for anaerobic digestate of organic fraction of municipal solid waste: Current status and future prospects. Waste Manag Res. 2019;37(1):27–39. 30761956 10.1177/0734242X18816793

[138] Bianco F, Race M, Papirio S, et al. Removal of polycyclic aromatic hydrocarbons during anaerobic biostimulation of marine sediments. Sci Total Environ. 2020;709:136141. 31887522 10.1016/j.scitotenv.2019.136141

[139] Van Poucke R, Egene CE, Allaert S, et al. Application of biochars and solid fraction of digestate to decrease soil solution Cd, Pb and Zn concentrations in contaminated sandy soils. Environ Geochem Health. 2020;42(6):1589–1600. DOI:10.1007/s10653-019-00475-4. 31776888

[140] Wang B, Gao B, Fang J. Recent advances in engineered biochar productions and applications. Crit Rev Environ Sci Technol. 2017;47(22):2158–2207.

[141] Qi X, Gou J, Chen X, et al. Application of mixed bacteria-loaded biochar to enhance uranium and cadmium immobilization in a co-contaminated soil. J Hazard Mater. 2021;401:123823. 33113745 10.1016/j.jhazmat.2020.123823

[142] Li G, Khan S, Ibrahim M, et al. Biochars induced modification of dissolved organic matter (DOM) in soil and its impact on mobility and bioaccumulation of arsenic and cadmium. J Hazard Mater. 2018;348:100–108. 29422192 10.1016/j.jhazmat.2018.01.031

[143] Kim H-B, Kim J-G, Kim T, et al. Mobility of arsenic in soil amended with biochar derived from biomass with different lignin contents: Relationships between lignin content and dissolved organic matter leaching. Chem Eng J. 2020;393:124687.

[144] Tang J, Cao C, Gao F, et al. Effects of biochar amendment on the availability of trace elements and the properties of dissolved organic matter in contaminated soils. Environ Technol Innov. 2019;16:100492.

[145] Bian R, Joseph S, Shi W, et al. Biochar DOM for plant promotion but not residual biochar for metal immobilization depended on pyrolysis temperature. Sci Total Environ. 2019;662:571–580. 30699377 10.1016/j.scitotenv.2019.01.224

[146] Wei J, Tu C, Yuan G, et al. Limited Cu (II) binding to biochar DOM: Evidence from C K-edge NEXAFS and EEM-PARAFAC combined with two-dimensional correlation analysis. Sci Total Environ. 2020;701:134919. 31726408 10.1016/j.scitotenv.2019.134919

[147] Fan Q, Sun J, Quan G, et al. Insights into the effects of long-term biochar loading on water-soluble organic matter in soil: Implications for the vertical co-migration of heavy metals. Environ Int. 2020;136:105439. 31918335 10.1016/j.envint.2019.105439

[148] Wang P, Peng H, Liu J, et al. Effects of exogenous dissolved organic matter on the adsorption–desorption behaviors and bioavailabilities of Cd and Hg in a plant–soil system. Sci Total Environ. 2020;728:138252. 32335403 10.1016/j.scitotenv.2020.138252

[149] Xing J, Xu G, Li G. Analysis of the complexation behaviors of Cu (II) with DOM from sludge-based biochars and agricultural soil: Effect of pyrolysis temperature. Chemosphere. 2020;250:126184. 32105854 10.1016/j.chemosphere.2020.126184

[150] Huang M, Li Z, Chen M, et al. Dissolved organic matter released from rice straw and straw biochar: Contrasting molecular composition and lead binding behaviors. Sci Total Environ. 2020;739:140378. 32758977 10.1016/j.scitotenv.2020.140378

[151] Kim H-B, Kim J-G, Choi J-H, et al. Photo-induced redox coupling of dissolved organic matter and iron in biochars and soil system: Enhanced mobility of arsenic. Sci Total Environ. 2019;689:1037–1043. 31466144 10.1016/j.scitotenv.2019.06.478

[152] Shaheen SM, Niazi NK, Hassan NE, et al. Wood-based biochar for the removal of potentially toxic elements in water and wastewater: a critical review. Int Mater Rev. 2019;64(4):216–247. DOI:10.1080/09506608.2018.1473096.

[153] Zhang B, Zhou S, Zhou L, et al. Pyrolysis temperature-dependent electron transfer capacities of dissolved organic matters derived from wheat straw biochar. Sci Total Environ. 2019;696:133895. 31465929 10.1016/j.scitotenv.2019.133895

[154] Yang F, Xu Z, Yu L, et al. Kaolinite enhances the stability of the dissolvable and undissolvable fractions of biochar via different mechanisms. Environ Sci Technol. 2018;52(15):8321–8329. DOI:10.1021/acs.est.8b00306. 29944830

[155] Cheng S, Chen T, Xu W, et al. Application research of biochar for the remediation of soil heavy metals contamination: a review. Molecules. 2020;25(14):3167. DOI:10.3390/molecules25143167. PMC7397277 32664440

[156] Li H, Dong X, Da Silva EB, et al. Mechanisms of metal sorption by biochars: biochar characteristics and modifications. Chemosphere. 2017;178:466–478. 28342995 10.1016/j.chemosphere.2017.03.072

[157] Sun Y, Xiong X, He M, et al. Roles of biochar-derived dissolved organic matter in soil amendment and environmental remediation: A critical review. Chem Eng J. 2021;424:130387.

[158] Du Z, He Y, Fan J, et al. Predicting apparent singlet oxygen quantum yields of dissolved black carbon and humic substances using spectroscopic indices. Chemosphere. 2018;194:405–413. 29223811 10.1016/j.chemosphere.2017.11.172

[159] Liu C-M, Diao Z-H, Huo W-Y, et al. Simultaneous removal of Cu^2+^ and bisphenol A by a novel biochar-supported zero valent iron from aqueous solution: synthesis, reactivity and mechanism. Environ Pollut. 2018;239:698–705. 29715689 10.1016/j.envpol.2018.04.084

[160] Wang A, Zheng Z, Li R, et al. Biomass-derived porous carbon highly efficient for removal of Pb (II) and Cd (II). Green Energy Environ. 2019;4(4):414–423. DOI:10.1016/j.gee.2019.05.002.

[161] Diao ZH, Dong FX, Yan L, et al. Synergistic oxidation of bisphenol A in a heterogeneous ultrasound-enhanced sludge biochar catalyst/persulfate process: Reactivity and mechanism. J Hazard Mater. 2020;384:121385. 31606253 10.1016/j.jhazmat.2019.121385

[162] Diao ZH, Zhang WX, Liang JY, et al. Removal of herbicide atrazine by a novel biochar based iron composite coupling with peroxymonosulfate process from soil: Synergistic effect and mechanism. Chem Eng J. 2021;409:127684.

[163] Liu X, Yang L, Zhao H, et al. Pyrolytic production of zerovalent iron nanoparticles supported on rice husk-derived biochar: simple, in situ synthesis and use for remediation of Cr (VI)-polluted soils. Sci Total Environ. 2020;708:134479. 31796288 10.1016/j.scitotenv.2019.134479

[164] Diao ZH, Dong FX, Yan L, et al. A new insight on enhanced Pb (II) removal by sludge biochar catalyst coupling with ultrasound irradiation and its synergism with phenol removal. Chemosphere. 2021;263:128287. 33297231 10.1016/j.chemosphere.2020.128287

[165] Malheiro C, Cardoso DN, Neves J, et al. Biochar in soil mitigates dimethoate hazard to soil pore water exposed biota. J Hazard Mater. 2020;400:123304. 32947708 10.1016/j.jhazmat.2020.123304

[166] Liu J, Jiang J, Meng Y, et al. Preparation, environmental application and prospect of biochar-supported metal nanoparticles: A review. J Hazard Mater. 2020;388:122026. 31958612 10.1016/j.jhazmat.2020.122026

[167] Tang F, Xu Z, Gao M, et al. The dissipation of cyazofamid and its main metabolite in soil response oppositely to biochar application. Chemosphere. 2019;218:26–35. 30465972 10.1016/j.chemosphere.2018.11.094

[168] Yavari S, Sapari NB, Malakahmad A, et al. Degradation of imazapic and imazapyr herbicides in the presence of optimized oil palm empty fruit bunch and rice husk biochars in soil. J Hazard Mater. 2019;366:636–642. 30579230 10.1016/j.jhazmat.2018.12.022

[169] You X, Jiang H, Zhao M, et al. Biochar reduced Chinese chive (*Allium tuberosum*) uptake and dissipation of thiamethoxam in an agricultural soil. J Hazard Mater. 2020;390:121749. 31818655 10.1016/j.jhazmat.2019.121749

[170] Huang T, Ding T, Liu D, et al. Degradation of carbendazim in soil: effect of sewage sludge-derived biochars. J Agric Food Chem. 2020;68(12):3703–3710. DOI:10.1021/acs.jafc.9b07244. 32125839

[171] You X, Suo F, Yin S, et al. Biochar decreased enantioselective uptake of chiral pesticide metalaxyl by lettuce and shifted bacterial community in agricultural soil. J Hazard Mater. 2021;417:126047. 33992003 10.1016/j.jhazmat.2021.126047

[172] Hou L, Zhang L, Chen X, et al. The benefits of biochar: Enhanced cadmium remediation, inhibited precursor production of nitrous oxide and a short-term disturbance on rhizosphere microbial community. Environ Pollut. 2021;272:116040. 33280913 10.1016/j.envpol.2020.116040

[173] Khan SU. 1st Edition of Pesticides in the soil environment, Editor name R.J. Wakeman. Elsevier; 2016. eBook ISBN: 9781483257068.

[174] Wu C, Liu X, Wu X, et al. Sorption, degradation and bioavailability of oxyfluorfen in biochar-amended soils. Sci Total Environ. 2019;658:87–94. 30572218 10.1016/j.scitotenv.2018.12.059

[175] Zhou C, Heal K, Tigabu M, et al. Biochar addition to forest plantation soil enhances phosphorus availability and soil bacterial community diversity. For Ecol Manage. 2020;455:117635.

[176] Baldrian P. The known and the unknown in soil microbial ecology. FEMS Microbiology Ecology. 2019;95(2):fiz005. 10.1093/femsec/fiz005 30624643

[177] Ibrahim M, Li G, Tang Y-T, et al. “Biochar effects acidic soil remediation and *Brassica oleracea* L. toxicity—a case study in subtropical area of China.” Environ. Technol. Innov. 2021;23:101588.

[178] Mukome FND, Buelow MC, Shang J, et al. Biochar amendment as a remediation strategy for surface soils impacted by crude oil. Environ Pollut. 2020;265:115006. 32593903 10.1016/j.envpol.2020.115006

[179] Irfan M, Hussain Q, Khan KS, et al. Response of soil microbial biomass and enzymatic activity to biochar amendment in the organic carbon deficient arid soil: a 2-year field study. Arab J Geosci. 2019;12(3):95. DOI:10.1007/s12517-019-4239-x.

[180] Quan G, Fan Q, Sun J, et al. Characteristics of organo-mineral complexes in contaminated soils with long-term biochar application. J Hazard Mater. 2020;384:121265. 31581012 10.1016/j.jhazmat.2019.121265

[181] Chen H, Awasthi MK, Liu T, et al. Influence of clay as additive on greenhouse gases emission and maturity evaluation during chicken manure composting. Bioresour Technol. 2018;266:82–88. 29957294 10.1016/j.biortech.2018.06.073

[182] Zhang P, Huang P, Xu X, et al. Spectroscopic and molecular characterization of biochar-derived dissolved organic matter and the associations with soil microbial responses. Sci Total Environ. 2020;708:134619. 31791751 10.1016/j.scitotenv.2019.134619

[183] Wang Y, Ma Z, Wang X, et al. Effects of biochar on the growth of apple seedlings, soil enzyme activities and fungal communities in replant disease soil. Sci Hortic. 2019;256:108641.

[184] Yuan H-Z, Zhu Z-K, Wei X-M, et al. Straw and biochar strongly affect functional diversity of microbial metabolism in paddy soils. J Integr Agric. 2019;18(7):1474–1485. DOI:10.1016/S2095-3119(18)62102-1.

[185] Ali N, Khan S, Li Y, et al. Influence of biochars on the accessibility of organochlorine pesticides and microbial community in contaminated soils. Sci Total Environ. 2019;647:551–560. 30089277 10.1016/j.scitotenv.2018.07.425

[186] Ge X, Cao Y, Zhou B, et al. Biochar addition increases subsurface soil microbial biomass but has limited effects on soil CO_2_ emissions in subtropical moso bamboo plantations. Appl Soil Ecol. 2019;142:155–165.

[187] Qiu X, Zhou G, Zhang J, et al. Microbial community responses to biochar addition when a green waste and manure mix are composted: a molecular ecological network analysis. Bioresour Technol. 2019;273:666–671. 30528727 10.1016/j.biortech.2018.12.001

[188] Jansson JK, Hofmockel KS. Soil microbiomes and climate change. Nat Rev Microbiol. 2020;18(1):35–46. 31586158 10.1038/s41579-019-0265-7

[189] Blanco-Canqui H, Laird DA, Heaton EA, et al. Soil carbon increased by twice the amount of biochar carbon applied after 6 years: field evidence of negative priming. GCB Bioenergy. 2020;12(4):240–251. DOI:10.1111/gcbb.12665.

[190] Futa B, Oleszczuk P, Andruszczak S, et al. Effect of natural aging of biochar on soil enzymatic activity and physicochemical properties in long-term field experiment. Agronomy. 2020;10(3):449. DOI:10.3390/agronomy10030449.

[191] Soinne H, Keskinen R, Heikkinen J, et al. Are there environmental or agricultural benefits in using forest residue biochar in boreal agricultural clay soil? Sci Total Environ. 2020;731:138955. 32417473 10.1016/j.scitotenv.2020.138955

[192] Kalu S, Simojoki A, Karhu K, et al. Long-term effects of softwood biochar on soil physical properties, greenhouse gas emissions and crop nutrient uptake in two contrasting boreal soils. Agric Ecosyst Environ. 2021;316:107454.

[193] Liao H, Zheng C, Long J, et al. Effects of biochar amendment on tomato rhizosphere bacterial communities and their utilization of plant-derived carbon in a calcareous soil. Geoderma. 2021;396:115082.

[194] Liu H, Zhou Y, Qin S, et al. Distribution of heavy metal resistant bacterial community succession in cow manure biochar amended sheep manure compost. Bioresour Technol. 2021;335:125282. 34020875 10.1016/j.biortech.2021.125282

[195] Cheng J, Lee X, Tang Y, et al. Long-term effects of biochar amendment on rhizosphere and bulk soil microbial communities in a karst region, southwest China. Appl Soil Ecol. 2019;140:126–134.

[196] Chang J, Yang Q, Dong J, et al. Reduction in Hg phytoavailability in soil using Hg-volatilizing bacteria and biochar and the response of the native bacterial community. Microbiol Biotechnol. 2019;12(5):1014–1023. DOI:10.1111/1751-7915.13457. PMC6681405 31241863

[197] Simarani K, Azlan Halmi MF, Abdullah R. Short-term effects of biochar amendment on soil microbial community in humid tropics. Arch Agron Soil Sci. 2018;64(13):1847–1860.

[198] Xu M, Gao P, Yang Z, et al. Biochar impacts on phosphorus cycling in rice ecosystem. Chemosphere. 2019;225:311–319. 30884292 10.1016/j.chemosphere.2019.03.069

[199] B-x Z, Ding K, X-r Y, et al. Straw biochar increases the abundance of inorganic phosphate solubilizing bacterial community for better rape (*Brassica napus*) growth and phosphate uptake. Sci Total Environ. 2019;647:1113–1120. 30180320 10.1016/j.scitotenv.2018.07.454

[200] Xiaoping T, Lei W, Yahong H, et al. Responses of soil microbial community structure and activity to incorporation of straws and straw biochars and their effects on soil respiration and soil organic carbon turnover. Pedosphere. 2019;29(4):492–503. DOI:10.1016/S1002-0160(19)60813-1.

[201] Woolet J, Whitman T. Pyrogenic organic matter effects on soil bacterial community composition. Soil Biol Biochem. 2020;141:107678.

[202] Bong CP, Lim LY, Lee CT, Klemeš JJ, Ho CS, Ho WS, The characterisation and treatment of food waste for improvement of biogas production during anaerobic digestion – A review. J. Clean. Prod. 2018; 1545–1558.31293294

[203] Guo XX, Liu HT, Zhang J. The role of biochar in organic waste composting and soil improvement: a review. Waste Manage. 2020;102:884–899. 10.1016/j.wasman.2019.12.003 31837554

[204] Dias BO, Silva CA, Higashikawa FS, et al. Use of biochar as bulking agent for the composting of poultry manure: effect on organic matter degradation and humification. Bioresour Technol. 2010;101:1239–1246. 19796932 10.1016/j.biortech.2009.09.024

[205] Khan N, Clark I, Sánchez-Monedero MA, et al. Maturity indices in co-composting of chicken manure and sawdust with biochar. Bioresour Technol. 2014;168;245–251. 24666624 10.1016/j.biortech.2014.02.123

[206] Sánchez-García M, Alburquerque JA, Sánchez-Monedero MA, et al. Biochar accelerates organic matter degradation and enhances N mineralisation during composting of poultry manure without a relevant impact on gas emissions. Bioresour Technol. 2015;192:272–279. 26038333 10.1016/j.biortech.2015.05.003

[207] Zhang JN, Chen GF, Sun HF, et al. Straw biochar hastens organic matter degradation and produces nutrient-rich compost. Bioresour Technol. 2016;200:876–883. 26600456 10.1016/j.biortech.2015.11.016

[208] Vandecasteele B, Sinicco T, D’Hose T, et al. Biochar amendment before or after composting affects compost quality and N losses, but not P plant uptake. J Environ Manage. 2016;168:200–209. 26708650 10.1016/j.jenvman.2015.11.045

[209] Czekała W, Malińska K, Cáceres R, et al. Co-composting of poultry manure mixtures amended with biochar – The effect of biochar on temperature and C-CO_2_ emission. Bioresour Technol. 2016;200:921–927. 26609949 10.1016/j.biortech.2015.11.019

[210] Duan Y, Yang J, Guo Y, et al. Pollution control in biochar-driven clean composting: emphasize on heavy metal passivation and gaseous emissions mitigation. J Haza Mater. 2021;420:126635. 10.1016/j.jhazmat.2021.126635 34329093

[211] He XQ, Yin HJ, Han LJ, et al. Effects of biochar size and type on gaseous emissions during pig manure/wheat straw aerobic composting: insights into multivariate-micro scale characterization and microbial mechanism. Bioresour Technol. 2019;271:375–382. 30293033 10.1016/j.biortech.2018.09.104

[212] Awasthi MK, Wang Q, Ren X, et al. Role of biochar amendment in mitigation of nitrogen loss and greenhouse gas emission during sewage sludge composting. Bioresour Technol. 2016b;219:270–280. 27497088 10.1016/j.biortech.2016.07.128

[213] Awasthi MK, Li J, Kumar S, et al. Effects of biochar amendment on bacterial and fungal diversity for co-composting of gelatin industry sludge mixed with organic fraction of municipal solid waste. Bioresour Technol. 2017;246:214–223. 28747259 10.1016/j.biortech.2017.07.068

[214] Achinas S, Achinas V, Euverink GJW. Microbiology and biochemistry of anaerobic digesters: an overview. Bioreactors. 2020. p. 17–26. 10.1016/B978-0-12-821264-6.00002-4

[215] Awasthi SK, Joshi R, Dhar H, et al. Improving methane yield and quality via co-digestion of cow dung mixed with food waste. Bioresour Technol. 2018;251:259–263. 29287278 10.1016/j.biortech.2017.12.063

[216] Khoshnevisan B, Duan N, Tsapkos P, et al. A critical review on livestock manure biorefinery technologies: sustainability, challenges, and future perspectives. Renew Sust Energ Rev. 2021;135:110033.

[217] Wang GJ, Li Y, Sheng L, et al. A review on facilitating bio-wastes degradation and energy recovery efficiencies in anaerobic digestion systems with biochar amendment. Bioresour Technol. 2020;314:123777. 32665106 10.1016/j.biortech.2020.123777

[218] Shen Y, Linville JL, Urgun-Demirtas M, et al. Producing pipeline-quality biomethane via anaerobic digestion of sludge amended with corn stover biochar with in-situ CO_2_ removal. Appl Energy. 2015;158:300–309.

[219] Fagbohungbe MO, Herbert BMJ, Hurst L, et al. Impact of biochar on the anaerobic digestion of citrus peel waste. Bioresour Technol. 2016;216:142–149. 27236401 10.1016/j.biortech.2016.04.106

[220] Wei WW, Guo WS, Ngo HH, et al. Enhanced high-quality biomethane production from anaerobic digestion of primary sludge by corn stover biochar. Bioresour Technol. 2020;306:123159. 32182472 10.1016/j.biortech.2020.123159

[221] Jang HM, Choi Y-K KE. Effects of dairy manure-derived biochar on psychrophilic, mesophilic and thermophilic anaerobic digestions of dairy manure. Bioresour Technol. 2018;250:927–931. 29198415 10.1016/j.biortech.2017.11.074

[222] Indren M, Birzer CH, Kidd SP, et al. Effects of biochar parent material and microbial pre-loading in biochar-amended high-solids anaerobic digestion. Bioresour Technol. 2020;298:122457. 31862677 10.1016/j.biortech.2019.122457

[223] Qiu L, Deng YF, Wang F, et al. A review on biochar-mediated anaerobic digestion with enhanced methane recovery. Renewable Sustainable Energy Rev. 2019;115:109373.

[224] Lü F, Luo CH, Shao LM, et al. Biochar alleviates combined stress of ammonium and acids by firstly enriching *Methanosaeta* and then *Methanosarcina*. Water Res. 2016;90:34–43. 26724437 10.1016/j.watres.2015.12.029

[225] Qin Y, Wang H, Li X, et al. Improving methane yield from organic fraction of municipal solid waste (OFMSW) with magnetic rice-straw biochar. Bioresour Technol. 2017;245:1058–1066. 28946388 10.1016/j.biortech.2017.09.047

[226] Li Q, Xu M, Wang G, et al. Biochar assisted thermophilic co-digestion of food waste and waste activated sludge under high feedstock to seed sludge ratio in batch experiment. Bioresour Technol. 2018;249:1009–1016. 29145112 10.1016/j.biortech.2017.11.002

[227] Jiang Q, Zhang C, Wu P, et al. Algae biochar enhanced methanogenesis by enriching specific methanogens at low inoculation ratio during sludge anaerobic digestion. Bioresour Technol. 2021;338:125493. 34273625 10.1016/j.biortech.2021.125493

[228] Lim EY, Tian H, Chen YY, et al. Methanogenic pathway and microbial succession during start-up and stabilization of thermophilic food waste anaerobic digestion with biochar. Bioresour Technol. 2020;314:1237541. 10.1016/j.biortech.2020.123751 32619808

[229] Mcinerney MJ, Sieber JR, Gunsalus RP. Syntropy in anaerobic global carbon cycles. Curr Opin Biotech. 2009;20(6):623–632. 19897353 10.1016/j.copbio.2009.10.001 PMC2790021

[230] Martins G, Salvador AF, Pereira L, et al. Methane production and conductive materials: a critical review. Environ Sci Technol. 2018;52:10241–10253. 30118213 10.1021/acs.est.8b01913

[231] Ali I, Asim M, Khan TA. Low cost adsorbents for the removal of organic pollutants from wastewater. J Environ Manage. 2012;113:170–183. 23023039 10.1016/j.jenvman.2012.08.028

[232] Ahmed MB, Zhou JL, Ngo HH, et al. Single and competitive sorption properties and mechanism of functionalized biochar for removing sulfonamide antibiotics from water. Chem Eng J. 2017;311:348–358.

[233] Rajapaksha AU, Vithanage M, Zhang M, et al. Pyrolysis condition affected sulfamethazine sorption by tea waste biochars. Bioresour Technol. 2014;166:303–308. 24926603 10.1016/j.biortech.2014.05.029

[234] Chen TW, Luo L, Deng SH, et al. Sorption of tetracycline on H_3_PO_4_ modified biochar derived from rice straw and swine manure. Bioresour Technol. 2018;267:431–437. 30032057 10.1016/j.biortech.2018.07.074

[235] Huang DL, Wang X, Zhang C, et al. Sorptive removal of ionizable antibiotic sulfamethazine from aqueous solution by graphene oxide-coated biochar nanocomposites: influencing factors and mechanism. Chemosphere. 2017;186:414–421. 28802133 10.1016/j.chemosphere.2017.07.154

[236] Deng JM, Dong HR, Zhang C, et al. Nanoscale zero-valent iron/biochar composite as an activator for Fenton-like removal of sulfamethazine. Sep Purif Technol. 2018;202:130–137.

[237] Fu D, Chen Z, Xia D, et al. A novel solid digestate-derived biochar-Cu NP composite activating H2O2 system for simultaneous adsorption and degradation of tetracycline. Environ Pollut. 2017;221:301–310. 27916494 10.1016/j.envpol.2016.11.078

[238] Sayğılı H, Güzel F. High surface area mesoporous activated carbon from tomato processing solid waste by zinc chloride activation: process optimization, characterization and dyes adsorption. J Clean Prod. 2016;113:995–1004.

[239] Lonappan L, Rouissi T, Das RK, et al. Adsorption of methylene blue on biochar microparticles derived from different waste materials. Waste Manage. 2016;49:537–544. 10.1016/j.wasman.2016.01.015 26818183

[240] Kannan N, Sundaram MM. Kinetics and mechanism of removal of methylene blue by adsorption on various carbons-a comparative study. Dyes Pigm. 2001;51:25–40.

[241] Inyang M, Dickenson E. The potential role of biochar in the removal of organic and microbial contaminants from potable and reuse water: a review. Chemosphere. 2015;134:232–240. 25958252 10.1016/j.chemosphere.2015.03.072

[242] Chen BL, Zhou DD, Zhu LZ. Transitional adsorption and partition of nonpolar and polar aromatic contaminants by biochars of pine needles with different pyrolytic temperatures. Environ Sci Technol. 2008;42(14):5137–5143. 18754360 10.1021/es8002684

[243] Chen BL, Chen ZM. Sorption of naphthalene and 1-naphthol by biochars of orange peels with different pyrolytic temperatures. Chemosphere. 2009;76(1):127–133. 19282020 10.1016/j.chemosphere.2009.02.004

[244] Nguyen TH, Cho HH, Poster DL, et al. Evidence for a pore-filling mechanism in the adsorption of aromatic hydrocarbons to a natural wood char. Environ Sci Technol. 2007;41:1212–1217. 17593721 10.1021/es0617845

[245] Kleineidam S, Schüth C, Grathwohl P, et al. Solubility-normalized combined adsorption-partitioning sorption isotherms for organic pollutants. Environ Sci Technol. 2002;36(21):4689–4697. DOI:10.1021/es010293b. 12433183

[246] Zhou Y, Cao SR, Xi CX, et al. A novel Fe_3_O_4_/graphene oxide/citrus peel-derived bio-char based nanocomposite with enhanced adsorption affinity and sensitivity of ciprofloxacin and sparfloxacin. Bioresour Technol. 2019;292:121951. 31400654 10.1016/j.biortech.2019.121951

[247] Sun K, Jin J, Keiluweit M, et al. Polar and aliphatic domains regulate sorption of phthalic acid esters (PAEs) to biochars. Bioresour Technol. 2012;118:120–127. 22705514 10.1016/j.biortech.2012.05.008

[248] Islam MS, Kwak JH, Nzediegwu C, et al. Biochar heavy metal removal in aqueous solution depends on feedstock type and pyrolysis purging gas. Environ Pollut. 2021;281:117094. 33848767 10.1016/j.envpol.2021.117094

[249] Qiu BB, Tao XD, Wang H, et al. Biochar as a low-cost adsorbent for aqueous heavy metal removal: a review. J Anal Appl Pyrol. 2021;155:105081.

[250] Xu XY, Cao XD, Zhao L. Comparison of rice husk- and dairy manure-derived biochars for simultaneously removing heavy metals from aqueous solutions: Role of mineral components in biochars. Chemosphere. 2013;92(8):955–961. 23591132 10.1016/j.chemosphere.2013.03.009

[251] Jeyasubramanian K, Thangagiri B, Sakthivel A, et al. A complete review on biochar: Production, property, multifaceted applications, interaction mechanism and computational approach. Fuel. 2021;292:120243.

[252] Caporale AG, Pigna M, Sommella A, et al. Effect of pruning-derived biochar on heavy metals removal and water dynamics. Biol Fert Soils. 2014;50(8):1211–1222. DOI:10.1007/s00374-014-0960-5.

[253] Bohli T, Villaescusa I, Ouederni A. Comparative study of bivalent cationic metals Adsorption Pb (II), Cd (II), Ni (II) and Cu (II) on olive stones chemically activated carbon. J Chem Eng Process Technol. 2013;4:100158.

[254] Inyang M, Gao B, Yao Y, et al. Removal of heavy metals from aqueous solution by biochars derived from anaerobically digested biomass. Bioresour Technol. 2012;110:50–56. 22325901 10.1016/j.biortech.2012.01.072

[255] Kim MS, Min HG, Koo N, et al. The effectiveness of spent coffee grounds and its biochar on the amelioration of heavy metals-contaminated water and soil using chemical and biological assessments. J Environ Manage. 2014;146:124–130. 25242543 10.1016/j.jenvman.2014.07.001

[256] Mohan D, Pittman CU, Bricka M, et al. Sorption of arsenic, cadmium, and lead by chars produced from fast pyrolysis of wood and bark during bio-oil production. J Colloid Interf Sci. 2007;310(1):57–73. DOI:10.1016/j.jcis.2007.01.020. 17331527

[257] Zhu J, Pigna M, Cozzolino V, et al. Competitive sorption of copper (II), chromium (III) and lead (II) on ferrihydrite and two organomineral complexes. Geoderma. 2010;159:409–416.

[258] Yang D, Wang L, Li ZT, et al. Simultaneous adsorption of Cd(II) and As(III) by a novel biochar-supported nanoscale zero-valent iron in aqueous systems. Sci Total Environ. 2020;708:134823. 31780167 10.1016/j.scitotenv.2019.134823

[259] Sr K, Manning B, Charlet L, et al. Removal of arsenic (III) from groundwater by nanoscale zero-valent iron. Environ Sci Technol. 2005;39(5):1291–1298. DOI:10.1021/es048991u. 15787369

[260] Li ZT, Wang L, Meng J, et al. Zeolite-supported nanoscale zero-valent iron: new findings on simultaneous adsorption of Cd (II), Pb (II), and As (III) in aqueous solution and soil. J Hazard Mater. 2018;344:1–11. 29028493 10.1016/j.jhazmat.2017.09.036

[261] Jia Y, Zhang YS, Fu JG, et al A novel magnetic biochar/MgFe-layered double hydroxides composite removing Pb^2+^ from aqueous solution: isotherms, kinetics and thermodynamics. Colloid. Surface. A Physicochem. Eng. Asp. 2019; 567: 278–287. 10.1016/j.colsurfa.2019.01.064

[262] Varjani S, Kumar G, Rene ER. Developments in biochar application for pesticide remediation: current knowledge and future research directions. J Environ Manage. 2019;232:505–513. 30502618 10.1016/j.jenvman.2018.11.043

[263] Tatarková V, Hiller E, Vaculík M. Impact of wheat straw biochar addition to soil on the sorption, leaching, dissipation of the herbicide (4-chloro-2-methylphenoxy) acetic acid and the growth of sunflower (*Helianthus annuus* L.). Ecotox Environ Safe. 2013;92:215–221. 10.1016/j.ecoenv.2013.02.005 23474069

[264] Cao XD, Harris W. Properties of dairy-manure-derived biochar pertinent to its potential use in remediation. Bioresour Technol. 2010;101:5222–5228. 20206509 10.1016/j.biortech.2010.02.052

[265] Wang HL, Lin KD, Hou ZN, et al. Sorption of the herbicide terbuthylazine in two New Zealand forest soils amended with biosolids and biochars. J Soil Sediment. 2010;10(2):283–289. DOI:10.1007/s11368-009-0111-z.

[266] Yu XY, Mu CL, Gu C, et al. Impact of woodchip biochar amendment on the sorption and dissipation of pesticide acetamiprid in agricultural soils. Chemosphere. 2011;85(88):1284–1289. DOI:10.1016/j.chemosphere.2011.07.031. 21862101

[267] Yu XY, Ying GG, Kookana RS. Reduced plant uptake of pesticides with biochar additions to soil. Chemosphere. 2009;76:665–671. 19419749 10.1016/j.chemosphere.2009.04.001

[268] Zhang P, Sun HW, Min LJ, et al. Biochars change the sorption and degradation of thiacloprid in soil: insights into chemical and biological mechanisms. Environ Pollut. 2018;236:158–167. 29414336 10.1016/j.envpol.2018.01.030

[269] Awasthi MK, Zhang Z, Wang Q, et al. New insight with the effects of biochar amendment on bacterial diversity as indicators of biomarkers support the thermophilic phase during sewage sludge composting. Bioresour Technol. 2017;238:589–601. 28482285 10.1016/j.biortech.2017.04.100

[270] Amoah-Antwi C, Kwiatkowska-Malina J, Thornton SF, et al. Restoration of soil quality using biochar and brown coal waste: a review. Sci Total Environ. 2020;722:137852. 32213438 10.1016/j.scitotenv.2020.137852

[271] Panahi HKS, Dehhaghi M, Ok YS, et al. A comprehensive review of engineered biochar: production, characteristics, and environmental applications. J Clean Prod. 2020;270:122462.

[272] Émg L, Reis MM, Frazão LA, et al. Biochar increases enzyme activity and total microbial quality of soil grown with sugarcane. Environ Technol Inno. 2021;21:101270.

[273] Wang XB, Song DL, Liang GQ, et al. Maize biochar addition rate influences soil enzyme activity and microbial community composition in a fluvo-aquic soil. Appl Soil Ecol. 2015c;96:265–272.

[274] Wang N, Chang ZZ, Xue XM, et al. Biochar decreases nitrogen oxide and enhances methane emissions via altering microbial community composition of anaerobic paddy soil. Sci Total Environ. 2017;581-582:689–696. 28063654 10.1016/j.scitotenv.2016.12.181

[275] Wong JTF, Chen Z, Chen X, et al. Soil-water retention behavior of compacted biochar-amended clay: a novel landfill final cover material. J Soil Sediment. 2017;17:590–598.

[276] Dick RP, Kandeler E. Encyclopedia of soils in the environment. Earth Environ Sci. 2005;448–456.

[277] Li Q, Song XZ, Yrjälä K, et al. Biochar mitigates the effect of nitrogen deposition on soil bacterial community composition and enzyme activities in a *Torreya grandis* orchard. Forest Ecol Manage. 2020;457:117717.

[278] Lammirato C, Miltner A, Kaestner M. Effects of wood char and activated carbon on the hydrolysis of cellobiose by β-glucosidase from *Aspergillus niger*. Soil Biol Biochem. 2011;43(9):1936–1942.

[279] Wen EG, Yang X, Chen HB, et al. Iron-modified biochar and water management regime-induced changes in plant growth, enzyme activities, and phytoavailability of arsenic, cadmium and lead in a paddy soil. J Hazard Mater. 2021;407:124344. 33162240 10.1016/j.jhazmat.2020.124344

[280] Zhu XM, Chen BL, Zhu LZ, et al. Effects and mechanisms of biochar-microbe interactions in soil improvement and pollution remediation: a review. Environ Pollut. 2017;227:98–115. 28458251 10.1016/j.envpol.2017.04.032

[281] Zimmerman AR, Ahn MY. Organo-mineral enzyme interactions and influence on soil enzyme activity. In: Shukla G., Varma A. (eds) Soil Biol Biochem. Berlin, Heidelberg: Springer. 2010;271–292. 10.1007/978-3-642-14225-3_15

[282] Joseph S, Graber ER, Chia C, et al. Shifting paradigms: development of high-efficiency biochar fertilizers based on nano-structures and soluble components. Carbon Manage. 2013;4:323–343.

[283] Quilliam RS, Glanville HC, Wade SC, et al. Life in the ‘charosphere’ - does biochar in agricultural soil provide a significant habitat for microorganisms?. Soil Biol. Biochem. 2013;65:287–293.

[284] Abel S, Peters A, Trinks S, et al. Impact of biochar and hydrochar addition on water retention and water repellency of sandy soil. Geoderma. 2013;202-203:183–191.

[285] Gao X, Cheng HY, Valle ID, et al. Charcoal disrupts soil microbial communication through a combination of signal sorption and hydrolysis. Acs Omega. 2016;1:226–233. 29938248 10.1021/acsomega.6b00085 PMC6010303

[286] Masiello CA, Chen Y, Gao X, et al. Biochar and microbial signaling: production conditions determine effects on microbial communication. Environ Sci Technol. 2013;47:11496–11503. 24066613 10.1021/es401458s PMC3897159

[287] Stefaniuk M, Oleszczuk P. Addition of biochar to sewage sludge decreases freely dissolved PAHs content and toxicity of sewage sludge-amended soil. Environ Pollut. 2016;218:242–251. 27461750 10.1016/j.envpol.2016.06.063

[288] Yang X, Liu JJ, McGrouther K, et al. Effect of biochar on the extractability of heavy metals (Cd, Cu, Pb, and Zn) and enzyme activity in soil. Environ Sci Pollut Res. 2016;23:974–984. 10.1007/s11356-015-4233-0 25772863

[289] Peng Z, Zhao H, Lyu H, et al. UV modification of biochar for enhanced hexavalent chromium removal from aqueous solution. Environ Sci Pollut Res. 2018;25(11):10808–10819. DOI:10.1007/s11356-018-1353-3. 29396828

[290] Peter A, Chabot B, Loranger E. Enhanced activation of ultrasonic pre-treated softwood biochar for efficient heavy metal removal from water. J Environ Manage. 2021;290:112569. 33865155 10.1016/j.jenvman.2021.112569

[291] Wang HY, Gao B, Wang SS, et al. Removal of Pb (II), Cu (II), and Cd (II) from aqueous solutions by biochar derived from KMnO_4_ treated hickory wood. Bioresour Technol. 2015a;197:356–362. 26344243 10.1016/j.biortech.2015.08.132

[292] Wang S, Gao B, Zimmerman AR, et al. Removal of arsenic by magnetic biochar prepared from pinewood and natural hematite. Bioresour Technol. 2015b;175:391–395. 25459847 10.1016/j.biortech.2014.10.104

[293] Wang Y, Liu R. H_2_O_2_ treatment enhanced the heavy metals removal by manure biochar in aqueous solutions. Sci Total Environ. 2018;628-629:1139–1148. 30045537 10.1016/j.scitotenv.2018.02.137

[294] Prodromou M, Pashalidis I. Copper (II) removal from aqueous solutions by adsorption on non-treated and chemically modified cactus fibers. Water Sci Technol. 2013;68:2497–2504. 24334902 10.2166/wst.2013.535

[295] Kwak J-H, Islam MS, Wang S, et al. Biochar properties and lead (II) adsorption capacity depend on feedstock type, pyrolysis temperature, and steam activation. Chemosphere. 2019;231:393–404. 31146131 10.1016/j.chemosphere.2019.05.128

[296] Son EB, Poo KM, Chang JS, et al. Heavy metal removal from aqueous solutions using engineered magnetic biochars derived from waste marine macro-algal biomass. Sci Total Environ. 2018;615:161–168. 28964991 10.1016/j.scitotenv.2017.09.171

[297] Wang HY, Gao B, Fang JN, et al. Engineered biochar derived from eggshell-treated biomass for removal of aqueous lead. Ecol Eng. 2018;121:124–129.

[298] Nadarajah K, Bandala ER, Zhang ZY, et al. Removal of heavy metals from water using engineered hydrochar: kinetics and mechanistic approach. J Water Process Eng. 2021;40:101929.

[299] Yang F, Zhang SS, Sun YQ, et al. Fabrication and characterization of hydrophilic corn stalk biochar-supported nanoscale zero-valent iron composites for efficient metal removal. Bioresour Technol. 2018;265:490–497. 29940499 10.1016/j.biortech.2018.06.029

[300] Agrafioti E, Kalderis D, Diamadopoulos E. Ca and Fe modified biochars as adsorbents of arsenic and chromium in aqueous solutions. J Environ Manage. 2014;146:444–450. 25199600 10.1016/j.jenvman.2014.07.029

[301] Deng JQ, Liu YG, Liu SB, et al. Competitive adsorption of Pb (II), Cd (II) and Cu (II) onto chitosan-pyromellitic dianhydride modified biochar. J Colloid Interf Sci. 2017;506:355–364. 10.1016/j.jcis.2017.07.069 28750237

[302] Yan T, Xue J, Zhou Z, et al. Biochar-based fertilizer amendments improve the soil microbial community structure in a karst mountainous area. Sci. Total Environ. 2021; 794(10):148757. 10.1016/j.scitotenv.2021.148757 34225142

[303] Ambaye TG, Rene ER, Dupont C, et al. Anaerobic digestion of fruit waste mixed with sewage sludge digestate biochar: influence on biomethane production. Front Energy Res. 2020;8:31.

[304] Liu M, Liu C, Liao W, et al. Impact of biochar application on gas emissions from liquid pig manure storage. Sci Total Environ. 2021;771:145454. 33736144 10.1016/j.scitotenv.2021.145454

[305] Chung WJ, Chang SW, Chaudhary DK, et al. Effect of biochar amendment on compost quality, gaseous emissions and pathogen reduction during in-vessel composting of chicken manure. Chemosphere. 2021;283:131129. 34153920 10.1016/j.chemosphere.2021.131129

[306] Zhang Q, Zhang X, Duan P, et al. The effect of long-term biochar amendment on N_2_O emissions: Experiments with N^15^-O^18^ isotopes combined with specific inhibition approaches. Sci Total Environ. 2021;769:144533. 33482542 10.1016/j.scitotenv.2020.144533

[307] Ji B, Chen J, Mei J, et al. Roles of biochar media and oxygen supply strategies in treatment performance, greenhouse gas emissions, and bacterial community features of subsurface-flow constructed wetlands. Bioresour Technol. 2020;302:122890. 32014728 10.1016/j.biortech.2020.122890

[308] Wang C, Chen D, Shen J, et al. Biochar alters soil microbial communities and potential functions 3-4 years after amendment in a double rice cropping system. Agric Ecosyst Environ. 2021;311:107291.

[309] Zhou Y, Awasthi SK, Liu T, et al. Patterns of heavy metal resistant bacterial community succession influenced by biochar amendment during poultry manure composting. J Hazard Mater. 2021;420:126562. 34252662 10.1016/j.jhazmat.2021.126562

[310] Awasthi SK, Duan Y, Liu T, et al. Can biochar regulate the fate of heavy metals (Cu and Zn) resistant bacteria community during the poultry manure composting? J Hazard Mater. 2021;406:124593. 33316669 10.1016/j.jhazmat.2020.124593

[311] Ren T, Gao W, Xu C, et al. Novel approaches of regulating soil micro-ecological environment based on modified biochar in plastic greenhouse. Environ Technol Innov. 2021;23:101740.

[312] Qian W, Liang J-Y, Zhang W-X, et al. A porous biochar supported nanoscale zero-valent iron material highly efficient for the simultaneous remediation of cadmium and lead contaminated soil. J Environ Sci. 2022;113:231–241. 10.1016/j.jes.2021.06.014 34963531

